# Dissecting conserved molecular mechanisms of biological toxin activity through CRISPR screening

**DOI:** 10.1042/BST20250099

**Published:** 2026-05-28

**Authors:** Matthew A. Waller, Tian Y. D'Araujo, Christopher E. Denes, G. Gregory Neely

**Affiliations:** Dr. John and Anne Chong Lab for Functional Genomics, Charles Perkins Centre, School of Life and Environmental Sciences, University of Sydney, Camperdown, NSW, Australia

**Keywords:** convergence, CRISPR screening, toxin, venom

## Abstract

Toxins, substances that are produced by living organisms with the potential to cause harm, demonstrate great diversity in their structure, function, and origin. Though some toxins have been repurposed for use as novel therapeutics, research tools, or for application in agriculture, the mechanism of action for many toxins remains uncharacterised. Pooled CRISPR screens offer a high-throughput and unbiased method for rapid annotation of the host cell genome and identification of factors mediating or modifying intoxication. In this review, we provide a brief overview of CRISPR screening before detailing how screens have been used to characterise toxins from various biological kingdoms. We highlight certain cell entry factors and intracellular processes as conserved targets of various toxins. Finally, we highlight limitations in the methods of CRISPR screens used thus far and make recommendations as to how screen design can be modified to more completely characterise toxin activity and elucidate systemic effects of intoxication.

## Introduction

Toxins have evolved over hundreds of millions of years and across kingdoms of life for both predation and protection [1]. These biological compounds demonstrate incredible functional diversity and have been of keen interest in drug discovery efforts owing to their potency, target specificity, and the range of phenotypes they can induce. Toxin characterisation has yielded a variety of medicines, with famous examples including captopril [2], a synthetic analogue of bradykinin-potentiating peptides isolated from the South American pit viper *Bothrops jararaca* and used for treatment of hypertension and cardiovascular conditions, and the bacteria-derived botulinum toxin (‘Botox’), which is used both therapeutically and cosmetically [3]. Furthermore, toxinological research has been instrumental in informing strategies for treatment of envenoming events and bacterial infections, development of critical tools for molecular biology research (such as ion channel inhibitors and activators) [4,5], or as biosensors for detection of pathogens in food and waste [6]. Advantageous use of toxins has also been observed in agriculture, specifically in their repurposing as pesticides [7]. However, the underlying mechanism of action for many of these toxins is poorly characterised.

The implementation of CRISPR–Cas systems has revolutionised molecular biology, democratising gene manipulation by offering robust and targeted genome perturbation at low cost and with ease of execution. Consequently, the application of CRISPR–Cas technology to targeted or whole-genome pooled screening has enabled functional annotation of the genome at a rate not previously possible. This review will provide an overview of the current state of the field of CRISPR screening with a focus on understanding the molecular mechanisms of single toxins as well as venoms as mixtures of toxins. We highlight key studies categorised by the kingdom of life from which the toxin originated, identifying convergent cell entry targets or mechanisms, as well as intracellular processes that control toxin/venom outcomes. Though toxin CRISPR screening is a nascent field, the ability to identify commonalities in the mechanism of action of various toxins permits the identification of small panels of pharmacological inhibitors that can be used in rapidly ascertaining the function of other novel toxins via drug screening, as useful tools in xenobiotic target discovery or may inform development of potential broad-spectrum therapeutics. Finally, we outline the limitations of current approaches to CRISPR screening in toxinology and make recommendations for improved future study designs.

## A brief primer: What is CRISPR screening?

CRISPR systems are typically composed of an effector protein, such as the Cas9 endonuclease, and an ∼20 nucleotide RNA molecule referred to as a single guide RNA (sgRNA) [8–12]. It is most widely used for gene knockouts (KO) where co-delivery of these components and homology-directed binding of the sgRNA–Cas9 complex to the locus of interest by sgRNA sequence complementarity induces a Cas9-mediated double-stranded break (DSB). Repair of this DSB by the cellular error-prone non-homologous end joining pathway results in insertions or deletions of nucleotides, causing frameshift errors that prematurely terminate protein expression. This is referred to as CRISPR knockout (CRISPRko). Modifications to the CRISPR–Cas9 system permit its use for various modes of gene manipulation. Fusion of different effector proteins to a catalytically dead Cas9 protein has been used to generate CRISPR machinery for targeted knockdown (CRISPR interference; CRISPRi) or upregulation (CRISPR activation; CRISPRa) of gene expression [13–20], cytosine and adenine base editors [21], and ‘prime editors’, capable of a broad range of targeted nucleotide edits and sequence insertions or deletions [22].

**Figure 1:**
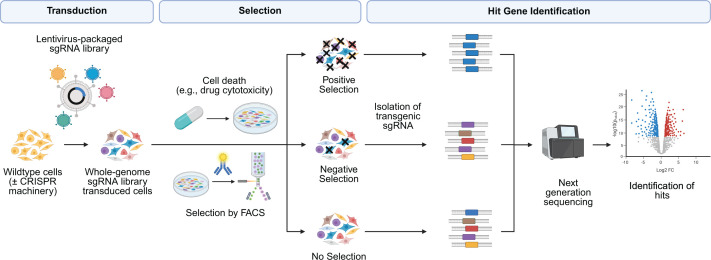
Conventional pooled CRISPR screening workflow Cells expressing the chosen CRISPR system are transduced with virus-packaged sgRNA library, frequently lentivirus. Library cells then undergo phenotypic selection, such as toxin-induced cell death or fluorescence-activated cell sorting (FACS). Screens may be designed to make use of positive selection, in which cells without the desired phenotype are removed from the population to leave only those with the desired phenotype. Alternatively, they may make use of negative selection, in which only cells with the desired phenotype are removed. Genomic DNA is then extracted from cells and integrated sgRNA cassettes are identified using NGS. Comparison of sgRNA abundance then allows for identification of ‘hit’ genes. Created in BioRender. Neely, G. (2026) https://BioRender.com/zw28m2e.

Pooled CRISPR screens are genetic screens in which cultured cells are mutagenised in bulk, resulting in simultaneous gene perturbations (up to whole genome scale) in a single population of cells, and then screened for a phenotype of interest (Figure 1). The CRISPR screening pipeline and key design considerations have been thoroughly reviewed elsewhere [23–25]. Typically, a collection of sgRNA sequences referred to as a ‘library’ is packaged into lentivirus. The cell line of interest is then transduced with the lentivirus sgRNA library at a low multiplicity of infection such that only a single sgRNA is expressed in each cell. The mutagenised cell pool then undergoes a phenotypic selection step that permits the enrichment or isolation of cells from the pooled population whose genomic modification confers a phenotype of interest. In most high-throughput screens, next-generation sequencing (NGS) of genomic DNA following PCR amplification or single-cell sequencing is used to identify individual sgRNAs responsible for the selected trait. Subsequent bioinformatic analysis tools then identify enriched or depleted sgRNA whose activity contributed to their phenotypic selection.

## Application of CRISPR screening to toxins

CRISPR screens have been used to characterise the mechanisms of action of toxins isolated from both bacteria and eukaryotes. Here, we provide an overview of screens investigating mechanisms of intoxication by bacterial toxins, plant toxins (phytotoxins), fungal toxins (mycotoxins), and animal venoms/toxins. Selected screens are highlighted in Table 1. A complete list of toxin CRISPR screens considered for this review can be found in Supplementary Table S1.

**Table 1:** Notable CRISPR screens of toxins originating from various kingdoms

Toxin	CRISPR modality	Cell line	Library	Selection	Notable hit genes	Notable pathways or processes
Bacterial toxins						
*Escherichia coli* Shiga toxin [26]	CRISPRko	HeLa (Human)	GeCKOv2	2 ng/ml Shiga toxin for 72 h	*SPTSSA, UGCG, B4GALT5, SPTLC2, A4GALT, SPTLC1, CERS2, LAPTM4A, TM9SF2, AHR*	Gb3 biosynthetic pathway, sphingolipid synthesis
*Escherichia coli* α-haemolysin [27]	CRISPRko	IMCD-3 (Mouse)	Brie	3 rounds of selection with 10 haemolytic units of α-haemolysin for 2 h (∼60% cell death) then recovery for 48 h	*Aagab, Ap2s1, Ap2m1, Armh3m Cltc, Cyfip1, Ldlr, Nckap1, Npc1, Pi4kb, Smdt1, Vps11*	Clathrin-mediated endocytosis, vesicle transport
*Photorhabdus luminescens* W14 Tc toxin (PTC3^W14^) [28]	CRISPRko	HeLa (Human)	GeCKOv2	3 rounds of selection with PTC3^W14^ (at increasing concentrations of 5, 10, and 20 nM) for 24 h	*MGAT2, MGAT1, MAN1A2, ACTG1*	N-glycan synthesis, sulfated glycosaminoglycans (GAGs)
*Staphylococcus aureus* α-haemolysin [29]	CRISPRko	U937 (Human)	GeCKOv2	0.5 μg/ml α-haemolysin for 7 days	*ADAM10, SYS1, ARFRP1, TSPAN14, SGMS1*	Sphingomyelin synthesis, intracellular trafficking
*Streptococcus intermedius* intermedilysin [30]	CRISPRko	HAP1 (Human)	TKOv3	2 ng/ml intermedilysin for 1 h, two weeks for recovery, then 10 ng/ml intermedilysin for 1 h	*CD59, SREBF2, LDLR, MOGS, PRKCSH, GANAB, MGAT1, SSR1, SSR2, SSR3, UGP2, GALE, UXS1, XYLT2, NDST1, EXT1, EXT2, B3GALT6, B4GALT7, UGCG, SLC35A2, TM9SF2*	GPI-anchor synthesis and attachment, nucleotide sugar synthesis, lipid and protein glycosylation; heparan sulfate pathway, cholesterol metabolism pathway, N-glycosylation, UDP-sugar synthesis, ganglioside synthesis
Plant toxins (Phytotoxins)						
*Dendrocnide excelsa* Excelsatoxin A (ExTxA) [31]	CRISPRko	TE-671 (Human)	TKOv3	3 rounds of co-incubation with 1 μM ExTxA, 5 μM veratridine, and 20 nM ouabain for 72 h	Protective: *SCN9A, TMEM233, RNF121, GPAA1, PIGT, CRELD1, PIGK, STT3B, PIGS, MMGT1, LMAN2L* Sensitising: *SPAG5, TMEM161B, NEDD4L*	Ubiquitination, N-glycosylation^*^, GPI anchor biosynthesis^*^
Hippeastrum hybrid lectin (HHL) [32]	(1) CRISPRko (2) CRISPRi (KRAB-dCas9)	A549 (Human)	(1) Custom KO library [34] (2) Custom CRISPRi sub-library (292 genes, based on KO screen hits and related genes)	(1) KO screen: Doxycycline treatment to induce XBP1s for 48 h, and then fixed and stained with FITC-labelled HHL. Sorted top and bottom 25% of HHL signal (2) CRISPRi screen: Cells either dox-treated or untreated for 48 h. Cell suspension then incubated with HHL-coupled magnetic beads and three rounds of magnetic separation performed	(1) CRISPRko screen: *MAN2A1, MGAT1, SLC35A2, WRB, MAN1A2, RMC1, ASNA1, TM9SF3, RAB7* (2) CRISPRi screen: Increased binding: *MAN1A1, MAN1A2, MGAT1, COG1-8, GET1, TM9SF3* Decreased binding: *CCDC22, CCDC93, VPS35L, LEO1, SMG7*	N-glycosylation
*Phaseolus vulgaris* leucoagglutinin (PHA-L) [33]	CRISPRko	HL-60 (Human)	Human GlycoGene sgRNA library	2 rounds of FACS: 20 min incubation of cell suspension with 10 μg/ml fluorescent PHA-L	Inhibiting: *MGAT2, MGAT5, MGAT1*	N-glycan biosynthesis
*Ricinus communis* ricin [34]	CRISPRko	K562 (Human)	Custom	4 rounds of selection with 0.25 ng/ml ricin toxin for 24 h then cells allowed to recover to normal doubling rate before next round	Protective: *DOLK, ALG14, ALG1, ALG2, ALG11, RFT1, ALG12, ALG5, ALG8, ALG10, ALG10B, OST4, MOQS, QANAB, MAN1B1, PMM2, GMPPS, GMDS, TSTA3, SLC35C1, SLC35A2, UGP2, GALE, MAN1A1, MGAT1, MAN2A1, MAN2A1, MGAT2, MGAT4B, MGAT5, FUTS, BUT4, B4GALT1* Sensitising: *MGAT3, ST3GAL2*	Nucleotide sugar synthesis, N-glycan synthesis pathway
*Ricinus communis* ricin [35]	CRISPRko	HeLa (Human)	GeCKOv2	4 rounds of selection with ricin (increasing concentrations of 0.2, 0.4, 0.8, and 1.5 ng/ml) for 48 h	*GOSR1, JTB, NBAS, TMEM165, TM9SF2, ALG5, ALG6, ALG8, MOGS, OST4, MAN1A2, MAN2A1, MGAT1, MGAT2, TSTA3, GMDS, SLC35C1, FUT4, VPS51, VPS51, VPS53, VPS54, GOSR1, NBAS, STX5, NAPG, ARL5B, ERP44, UBE2G2*	N-linked protein glycosylation, fucosylation, membrane trafficking, ER-associated protein degradation/quality-control pathways
Fungal toxins (Mycotoxins)						
*Amanita phalloides* α-amanitin [36]	CRISPRko	HAP1 (Human)	Brunello	1.5 μM α-amanitin (LD50) for 7 days	*TAF4, SLC46A3, MGAT1, SPPL3, WDR81, PSENEN, KIAA1033, STT3B, TIGD5, LAMTOR2*	Apoptosis, N-glycan biosynthesis, cholesterol metabolism, colorectal cancer, neurotrophin signalling pathway, necroptosis, sphingolipid signalling pathway
*Aspergillus spp.* aflatoxin B1 (AFB1) [37]	CRISPRko	PLC/PRF/5 (Human)	Brunello	6 rounds of 8 μM AFB1 for 48 h	*AHR, POR, KEAP1, SAFB, ALAS1, MYC, ONECUT1, WDR83, SLC5A5, RHEB*	Genotoxicity
*Aspergillus spp.*AFB1 [38]	CRISPRko	IPI-2I (Porcine)	Custom	3 rounds of AFB1, Increasing concentration from 5 μg/ml to 8 μg/ml	*CBS, INHBA*	Mitochondrial protein methylation, impaired mitochondrial function, oxidative stress, ubiquitination
*Candida albicans* candidalysin [39]	CRISPRko	TR146 (Human)	Brunello	5 rounds of 30 μM candidalysin for 6 h	*B3GALT6, TYK2, XYLT2, B3GAT3, SLC39A9, GBF1, EMP1*	GAG biosynthesis
*Fusarium spp.* fusaric acid [40]	CRISPRko	IPEC-J2 (Porcine)	Custom	50 μg/mL fusaric acid for at least 7 days (Until all control group cells died)	*TP53, MDH2, PDHB, G6PD, PDP1, CS, STOML2, LIAS, NDUFS4, USP28*	TCA cycle, pyruvate metabolism, carbon metabolism, glycolysis/gluconeogenesis
Animal toxins/venoms						
*Chironex fleckeri* venom [41]	CRISPRko	HAP1 (Human)	GeCKOv2	1 μg/ml *C. fleckeri* venom for 14 days	*ATP2B1*, *MBTPS1, MBTPS2, SCAP, SGMS1, SREBF2*	Regulation of cholesterol biosynthesis by SREBP (SREBF), apoptotic cleavage of cell adhesion proteins, other semaphorin interactions, Endosomal Sorting Complex Required for Transport, amino acid synthesis and interconversion (transamination), metalloprotease DUBs, regulation of TP53 activity through phosphorylation, signalling by retinoic acid, sphingolipid *de novo* biosynthesis
*Naja pallida* venom *Naja nigricollis* venom [42]	CRISPRko	HAP1 (Human)	TKOv3	5 μg/ml *N. pallida* or *N. nigricollis* venom for 9 days	*Naja pallida* Sensitising: *SMARCD1, CDK13, HDAC3, ZFAT, CRAMP1L* Protective: *TMEM50A, LEPROTL1, NDST1, XYLT2, EXT1, EXTL3, SLC35B2* *Naja nigricollis* Sensitising: *TSC1, TSC2, TBC1D7, SMARCC1, INPPL1, APPBP2* Protective: *LEPROTL1, TMEM50A, EXT1, B4GALT7, EXT2, EXTL3, XYLT2, NDST1, SLC35B2*	*N. pallida:* Heparan sulfate, GAG-protein linkage region, chondroitin sulfate, dermatan sulfate, heparan sulfate (late stages), aspartate degradation II, HIPPO signalling, PXR signalling, L-cysteine degradation III, thyroid hormone metabolism II *N. nigricollis:* Heparan sulfate, GAG-protein linkage region, chondroitin sulfate, dermatan sulfate, heparan sulfate (late stages), pre-mRNA, L-glutamine biosynthesis II, thyroid hormone metabolism II, molybdenum cofactor, BCKDC
*Hadronyche infensa* gomesin [43]	CRISPRko	MM96L (Human)	TKOv3	4 rounds of 25 μM HiGom (LD90) for 72 h followed by 72 h in regular culture media	Protective: *CISD1, SAP30BP, CCL5, EDNRA, CYBRD1, TM4SF20, UGP2, CMAS, ST3GAL5, B4GALT5, UGCG* Sensitising: *OPRL1, MIS18A, C4orf40*	CMP-N-acetylneuraminate biosynthesis I (Eukaryotes), GDP-mannose biosynthesis, sialic acid metabolism, colonic acid building blocks biosynthesis

* Indicates pathways or processes inferred from gene hits.

### Bacterial toxins

A systematic review conducted for the Global Burden of Disease Study 2019 found that 33 bacterial pathogens were associated with 7.7 million deaths, representing 13.6% of global deaths for that year [44]. Furthermore, antimicrobial-resistant bacteria accounted for 1.14 million deaths in 2021 [45]. The sustained impact of bacterial infection on global human health and the increasing threat of antimicrobial resistance support the use of systematic CRISPR screening to characterise host factors mediating bacterial infection and serve as a foundation for the development of novel antimicrobials or therapies. Among all toxin classes, bacterial toxins have been the most prevalently characterised by CRISPR screening.

Bacterial toxins may be broadly categorised according to their mechanism of action [46]. These categories include those damaging cell membranes, inhibiting protein synthesis, activating secondary messenger pathways, inhibiting neurotransmitter release, and stimulating host immune response.

Pore-forming toxins (PFTs), which are the most common bacterial toxins and exert their cytotoxicity by inserting themselves into the host cell membrane to drive ion flux between the intracellular and extracellular compartments [47], have been the focus of a number of CRISPR screens [27,29,30,48–54]. These screens have served both to re-confirm established mechanisms for PFT function such as dependence on plasma membrane cholesterol or use of specific receptors (i.e. use of CD59 by intermedilysin) and to yield novel insights of PFT–cell interactions. Notable examples include screens that highlighted the role of lipid rafts in PFT function. Virreira Winter et al. identified SYS1, ARFRP1, and TSPAN14 as regulators of *Staphylococcus aureus* α-hemolysin (αHL) receptor ADAM10 cell surface expression as well as SGMS1, whose KO minimally altered ADAM10 surface expression while impeding αHL cytotoxicity, leading them to hypothesise a lipid raft-dependent function [29]. This screen thereby positions SYS1, ARFRP1, TSPAN14, and SGMS1 as xenobiotic target genes for treatment of αHL intoxication via the modulation of ADAM10. Similarly, Sanduja et al. identified KO of glycosphingolipid biosynthetic genes* UGCG*, *B4GALT5*, and *GALE* to inhibit streptolysin O (SLO) cytotoxicity and demonstrated that SLO-binding to lipid rafts was facilitated by glycosphingolipids [54]. Another notable screen was that of Drabavicius et al., who found intermedilysin activity to be dependent not only on known receptor CD59 and cholesterol, but also N-glycosylation, UDP-sugar synthesis, heparan sulfates, and glucosyl- and/or lactosylceramides [30], implicating a range of cell surface factors in mediating intermedilysin binding and pore formation. Notably, KO of heparan sulfate pathway genes only modestly improved IC_50_, though these KOs and treatment with heparinase slowed pore formation while supply of exogenous heparin was protective. It was hypothesised that interactions between intermedilysin and heparan sulfate increase its chance of binding CD59. Ganglioside biosynthesis hit genes such as *UGCG* and *SLC35A2* were validated but genes downstream of the synthesis of lactosylceramide were not identified as hits, suggesting that intermedilysin activity may be dependent on lactosylceramide- and glucosylceramide-mediated membrane organisation. In one of the more extreme examples of subverting conventional wisdom, Kuhn et al. identified that toxicity of *E. coli* αHL in intestinal epithelial cells is dependent on LDLR-mediated endocytic uptake rather than cell surface pore formation [27]. Together, these examples demonstrate the ability of whole-genome CRISPR screening to provide detailed mechanistic characterisations of individual toxins even in cases where mechanism is already partially characterised.

Other bacterial toxin screens have identified a range of novel receptors used for cell entry, including LDLR for *Clostridium difficile* toxin A and *Clostridium novyi* alpha-toxin [55,56], LDL receptor related protein 1 (LRP1) for both *Pasteurella multocida* toxin and *C. difficile* toxin B [57,58], Frizzled proteins and TFPI for *C. difficile* toxin B [59–61], and semaphorins as receptors for *Paeniclostridium sordellii* lethal toxin [62,63]. Conversely, bacterial toxin screens have also highlighted the common use of cellular entry targets by toxins of different classes, clades, and species. These cell entry targets include N-glycans [28,64–67], GAGs [28,30,55,56,67], and cholesterol [30,54]. These examples highlight the utility of CRISPR screening in yielding novel insights and identifying conserved pathways for toxins of medically relevant bacteria.

### Plant toxins (phytotoxins)

Plants produce a range of toxins (phytotoxins) for defence against predators and pathogens. Human exposure to plant toxins can result in symptoms ranging from gastrointestinal disturbance, nausea, pain, or dermatological allergic reaction to organ damage or even death [68]. These toxins have in some cases been repurposed for use as medicines (i.e. as antioxidants and chemotherapeutics) or as pesticides. Classes of plant toxins include ribosomal inactivating proteins, lectins, plant protease inhibitors, α-amylase inhibitors, canatoxin-like proteins, ureases, arcelins, algogens, antimicrobial peptides, and PFTs [69].

Ricin, a ribosome-inactivating protein isolated from *Ricinus communis*, is the plant toxin that has received the most attention from pooled CRISPR screening approaches. As host cell genetic factors contributing to ricin mechanism of action had been thoroughly characterised by earlier RNA interference screens [70–72], ricin was used for the early development of CRISPR sgRNA libraries. Aiming to determine sgRNA design criteria for developing the first CRISPRi and CRISPRa libraries, Gilbert et al. used small sgRNA libraries tiling 49 different genes previously established as mediating ricin toxicity [70,73]. In validating these 49 genes, they re-confirmed the role of retrograde endosomal trafficking in ricin toxicity. Morgens et al. later performed CRISPRko screens for both growth and sensitivity to ricin to characterise Cas9 off-target activity and toxicity [34]. In addition to known ricin regulators, their screen identified 985 genes previously not associated with ricin activity, including those found in the nucleotide sugar and N-glycan biosynthesis pathways. Though subsequent validation was not performed as the primary motivation for this study was to investigate off-target Cas9 toxicity, these genes should mediate synthesis of cell surface glycans required for ricin entry and so represent a list of novel hits for xenobiotic target validation. Most recently, Tian et al. performed a CRISPRko screen for ricin that identified both novel and established genes associated with N-linked protein glycosylation, fucosylation, membrane trafficking and ER-associated protein degradation/quality control [35]. They also found two genes, *TMEM165* and *TM9SF2*, whose KO reduced global glycosphingolipid levels and inhibited toxicity of both ricin and Shiga toxins. With only limited overlap of screen hits, these studies highlight how screen outcomes vary largely depending on several critical design parameters, including choice of cell line (K562 or HeLa cells), CRISPR modality (CRISPRko, CRISPRi, and CRISPRa), choice of sgRNA library (custom libraries or the commercially available GeCKOv2 library) design of the phenotypic selection step (which varied in the concentration of ricin used, number of rounds of selection and the duration of treatment with ricin) and finally the analysis method and strategy for identifying ‘hits’.

CRISPR screening has also been used to characterise Excelsatoxin A (ExTxA), a ‘gympietide’ originating from the venom of Australian stinging nettle *Dendrocnide excelsa* and the first-identified Na_V_ channel-modulating toxin of plant origin [31]. As ExTxA was observed to inhibit Na_V_1.7 channel inactivation in certain neuronal cell lines but not others, it was suspected that an accessory protein was required for its activity. A CRISPRko screen was performed in human neuroblastoma TE-671 cells where ExTxA, the sodium channel activator veratridine and the sodium efflux inhibitor oubain were co-treated to induce cell death via cationic overload. TMEM233 was found to be necessary for the observed inhibition of Na_V_1.7 inactivation, binding ExTxA and directly interacting with Na_V_1.7. ExTxA therefore represents a novel tool for probing Na_V_1.7 dynamics with potential applications for treatment of a range of human ailments, including neurological and cardiovascular disorders. Its utility also implicates other gympietides as promising leads for drug repurposing and, through its interaction with TMEM233, suggests other members of the dispanin protein family may be leads for xenobiotic target discovery.

Other screens for plant toxins include a screen investigating glycogenes, which identified *MGAT1*, *MGAT2*, and *MGAT5* as necessary for synthesis of N-glycans used by *Phaseolus vulgaris* leucoagglutinin for cell surface binding (though not for O-glycan binding lectins *Vicia villosa* lectin or peanut agglutinin) [33]. Notably, *MGAT1* was also identified in CRISPR screens for regulating N-glycan biosynthesis under high mannose conditions and validated via binding of Hippeastrum hybrid lectin and Griffithsin, alongside *MAN1A2* and *MAN2A1* [32]. These studies suggest the genetic or pharmacologic inhibition of N-glycan synthesis may be broadly protective against various toxic lectins.

It is worth noting that there are a large number of poisonous plants frequently encountered by humans whose toxins have not yet been characterised by CRISPR screening and are good candidates for future research. These plants include oleander (*Nerium oleander*), foxglove (*Digitalis purpurea*), Angel’s trumpet (*Brugmansia spp.*), poison ivy (*Toxicodendron spp*.), and deadly nightshade (*Atropa belladonna*).

Notably, several classes of chemotherapeutics are derived from plants, including the taxanes, vinca alkaloids, podophyllotoxins, and camptothecins [74]. A considerable number of CRISPR screens have been performed to characterise the activity of these plant-derived chemotherapeutics. In one example, our lab performed 27 CRISPR screens for chemotherapeutics including plant-derived compounds vincristine, vinorelbine, docetaxel, irinotecan, camptothecin, and topotecan (as well as bacterial toxin analogues vorinostat and idarubicin, and sea sponge toxin analogues cytarabine and gemcitabine) [75]. These screens identified 56 chemotherapy resistance genes, including 10 multidrug resistance genes. One such gene, *c1orf115*, thereafter named *Required for Drug-induced Death*, inhibited death induced by vincristine, paclitaxel and vinorelbine, among other non-plant-derived chemotherapeutics. These screens are of vital importance to informing clinical use of plant-derived therapeutics, specifically for understanding mechanisms of drug resistance, as well as informing our understanding of how these toxins attack cells.

### Fungal toxins (mycotoxins)

Toxins produced by fungi, referred to as mycotoxins, are found in approximately 25% of the world’s food supply [76] and result in estimated mean economic losses of USD $ 932 million annually in the US alone [77]. Hundreds of different mycotoxins have been identified, with aflatoxins, ochratoxins, trichothecenes, zearalenone, fumonisins, and ergot alkaloids the classes of greatest significance to human health and agriculture [78]. Furthermore, exposure to mycotoxins may cause acute conditions like nausea, vomiting and loss of appetite, but can also be carcinogenic, neurotoxic or mutagenic [79]. Given their pervasiveness, diversity and the range of effects they have on human health, there is a clear need to thoroughly characterise the mechanism of action of these toxins and identify drugs/inhibitors that can block their function or tap into this rich resource for medical and/or research applications. To date, CRISPR screens have only been used to characterise a small number of mycotoxins.

Several CRISPR screens have focused on characterising the action of *Aspergillus spp*. AFB1, which has been identified as a causative agent for liver cancer. Zhu et al. carried out a CRISPRko screen in the human hepatocyte cell line PLC/PRF/5, identifying the KO of aryl hydrocarbon receptor (AHR) as protective against AFB1-induced cell death [37]. The study demonstrated direct binding of AFB1 to AHR and found AHR to mediate expression of cytochrome P450 genes whose function enables the formation of genotoxic AFB1–DNA adducts. AHR was also shown to mediate AFB1-induced accumulation of long chain fatty acids, which was correlated with increased expression of necroptosis markers. Zhang et al. performed a screen for escalating doses of AFB1-induced cytotoxicity in porcine kidney cells, characterising BTB and CNC homolog 1 (BACH1) as a transcriptional repressor whose KO rescues cells from AFB1-induced oxidative damage [80]. Most recently, Yang et al. identified *CBS* KO as protecting against AFB1-mediated inhibition of intra-mitochondrial protein methylation and decreases in global protein ubiquitination [38]. The identification of different regulators of AFB1 toxicity highlights the importance of screening in multiple physiologically relevant cell lines to fully elucidate toxin mechanisms of action and demonstrates interspecies variation in cellular response to toxins.

*Amanita phalloides*, colloquially known as death cap mushroom, is among the most toxic mushrooms in the world. *A. phalloides* produces α-amanitin, a highly toxic member of the cyclopeptide class of amatoxins that inhibits RNA polymerase II and causes subsequent cell death. It has been estimated that over 90% of mushroom fatalities are due to ingestion of amatoxin-containing mushrooms [81]. CRISPRko screening in the human HAP1 cell-line-identified dependence of α-amanitin toxicity on N-glycan biosynthesis and key regulator *STT3B* (as well as cholesterol metabolism), which was validated by pharmacological inhibition of N-glycan biosynthesis using kifunensine [36]. Indocyanine green (ICG) was identified by *in silico* screening of FDA-approved molecules as an STT3B inhibitor and subsequent validation assays confirmed ICG pretreatment inhibited α-amanitin-induced toxicity both *in vitro* and *in vivo*.

Other mycotoxin screens have: identified KO of GAG biosynthesis genes *XYLT2*, *B3GALT6*, and *B3GAT3* as inhibiting *Candida albicans* candidalysin activity, characterised the role of sulfated GAGs in enabling candidalysin cell entry and inhibited candidalysin activity using exogenous sulfated GAGs [39]; and posited KO of TCA cycle-related genes *MDH2* and *PDHB* as reducing the increase in ROS levels, caspase-3, and HIF-1a induced by fusaric acid, and demonstrated that *MDH2* KO protects against four other mycotoxins (deoxynivalenol, zearalenone, T-2 toxin, and fumonisin B1) [40]. Notably, targeting of mitochondrial function was observed in CRISPR screens for both AFB1 and fusaric acid.

### Animal toxins and venoms

Animal venoms have been defined as toxic secretions that manipulate the physiological functions of a living target organism through direct delivery [82,83]. With ∼100 lineages, and tens of thousands of species recognised as venomous, there is a great amount of variation and complexity [84]. Venoms are mixtures of proteins, peptides, and bioactive molecules, making them far more complex to understand than a singular toxin. CRISPR screens can therefore be a powerful tool in discovering mechanistic insights by which venoms act on the host. To date, three CRISPR screens have been reported on animal venoms/toxins and their human targets, all performed by our group. The first, a CRISPRko screen in HAP1 cells for box jellyfish (*Chironex fleckeri*) venom, found ATPase plasma membrane Ca^2+^ transporting 1 (ATP2B1), SGMS1, and key components of the SREBP pathway of cholesterol biosynthesis (SCAP, MBTPS1, or MBTPS2) to be involved in venom-induced cell death [41]. Importantly, cyclodextrins (MβCD and HPβCD) could be used to deplete cholesterol from the cell surface and rescue the pain and tissue necrosis associated with envenoming. Similarly, a CRISPRko screen for spitting cobra venom (*Naja pallida* and *Naja nigricollis*) identified numerous genes that could modify venom action, including many that regulate proteoglycan biosynthesis [42]. Aside from basic understanding of venom action, these findings allowed for the translation of the low molecular weight heparinoid tinzaparin as a therapeutic against the tissue damage caused by cobra venoms. We recently reported a CRISPRko screen in melanoma MM96L cells for gomesin, a toxin isolated from the venom of an Australian funnel web spider (*Hadronyche infensa*) [43]. This screen identified genes such as *ST3GAL5*, *B4GALT5*, and *UGCG* as mediating glycosphingolipid-dependent gomesin cytotoxicity in melanoma cells, with *ST3GAL5* KO being validated as inhibiting gomesin-mediated reduction in volume of MM96L xenograft melanoma tumours.

Screening of animal venoms is the most nascent category of toxin CRISPR screening. The small number of extant studies may be attributed to several factors, including the heterogeneous nature of venoms as cocktails of bioactive molecules. These mixtures raise questions of which is the causative toxin for an observed phenotype, and screening is further confounded by the possibility for toxins to be acting synergistically. These screens are also complicated by the considerable variability that exists within venoms even of the same species. It has been proposed that animal venom variation is dynamic; a result of the interplay of a range of factors including ontology and sex, ecological variables such as geography, temperature, and prey type, and genetics [85,86]. Furthermore, screening may be constrained by limited supply and/or lack of commercial availability of venoms, with venom extraction posing both technically difficult (e.g. venom extraction methods can alter component abundance) and hazardous to the researcher. Venomous species may also be cryptic or rare, may be difficult (or unfeasible) to keep in captivity, and some have been shown capable of varying not just the volume (‘venom metering’) but also composition of their venom [84]. Finally, choice of appropriate cell models can be difficult as envenoming is typically a multi-tissue or system-level insult. While the above examples highlight the utility of *in vitro* CRISPR screening in understanding the action of crude venoms at a cellular level, rigorous *in vitro* and *in vivo* validation is required to evaluate effects of envenomation at an organismal level.

## Convergence of toxin mechanism of action

**Figure 2:**
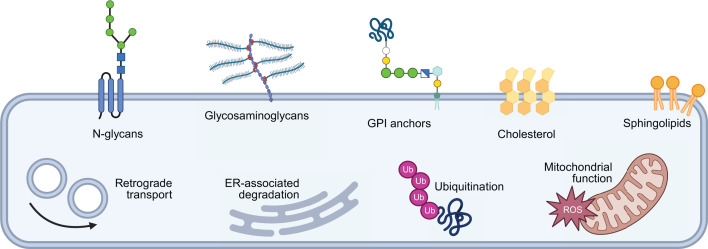
Conserved cell entry factors and intracellular processes targeted by toxins of diverse origins CRISPR screens have identified conserved host cell targets of toxins originating from various kingdoms of life. These include factors for cellular entry, such as N-glycans, GAGs, GPI anchors, cholesterol, and sphingolipids, and intracellular processes such as retrograde transport, ER-associated degradation, ubiquitination, and mitochondrial function. Created in BioRender. Neely, G. (2026) https://BioRender.com/b5navnd.

Evaluation of published toxin CRISPR screens highlights convergent mechanisms of cellular entry and intracellular processes targeted by toxins originating from various kingdoms of life (Table 2 and Figure 2). Common cellular entry targets include N-glycans, GAGs, GPI anchors, cholesterol, and sphingolipids. N-glycosylation serves several critical functions within cells, including mediating cell adhesion, receptor–ligand binding, and signalling at the cell surface as well as promoting protein folding and endoplasmic reticulum (ER) quality control [87]. GAGs, long negatively charged polysaccharides that are covalently bonded to proteins to form proteoglycans, play essential roles at the cell surface in mediating cellular adhesion, extracellular matrix interactions. and cell signalling [88,89]. Glycosylphosphatidylinositol (GPI) anchors function to tether proteins to the cell membrane, enabling their role in a vast array of functions [90]. In the context of the cellular membrane, cholesterol and sphingolipids serve as essential structural and functional components, providing rigidity to the fluid lipid-bilayer and forming lipid-rich microdomains referred to as lipid rafts [91,92]. They additionally play important roles in both intra- and intercellular signalling [93–96]. Targeting these entry factors, which are essential components of the cell membrane of all eukaryotic cells, gives toxins broad-spectrum activity against cells of diverse origins, increasing their utility in both defence and predation. Notably, O-glycans [30,65,67], LDLR, and LRP1 have been identified as cell entry factors targeted by multiple bacterial toxins but have not yet been validated in CRISPR screens for toxins originating from other kingdoms of life [27,55–58]. Many toxins also target specific membranous receptors, such as the use of Ca_V_3.1 by *Bordetella pertussis* dermonecrotic toxin [97], TMEM233 by ExTxA [31], and ATP2B1 by *Chironex fleckeri* venom [41]. These strategies of targeting both high-affinity receptors as well as lower affinity membrane constituents such as charged heparan sulfate proteoglycans is mirrored in viral infection [98], representing a general strategy of cellular attachment and entry used by exogenous biological agents.

**Table 2:** Cell surface factors and intracellular processes identified as conserved targets of toxins from across multiple kingdoms of life

Cellular target	Toxin class	Toxin
N-glycans	Bacterial	*Aeromonas hydrophila* proaerolysin [64]
		*Escherichia coli* subtilase cytotoxin [65]
		Lipopolysaccharide [66]
		*Photorhabdus luminescens* Tc toxin [28]
		*Streptococcus intermedius* intermedilysin [30]
	Plant	*Dendrocnide excelsa* Excelsatoxin A [31]
		Hippeastrum hybrid lectin [32]
		*Phaseolus vulgaris* leucoagglutinin [33]
		*Ricinus communis* ricin [34,35]
	Mycotoxin	*Amanita phalloides* α-amanitin [36]
		*Fusarium spp*. T-2 toxin [99]
GAGs	Bacterial	*Clostridium difficile* toxin A [55]
		*Clostridium novyi* alpha-toxin [56]
		*Morganella morganii* Tc toxin [67]
		*Photorhabdus luminescens* Tc toxin [28]
		*Streptococcus intermedius* intermedilysin [30]
	Mycotoxin	*Candida albicans* candidalysin [39]
	Animal venom	*Naja pallida* venom *Naja nigricollis* venom [42]
GPI anchors	Bacterial	*Aeromonas hydrophila* proaerolysin [64]
		*Clostroidies difficile* type 4 toxin B [60,61]
		*Clostridium septicum* alpha-toxin [100]
		*Streptococcus intermedius* intermedilysin [30,50]
	Plant	*Dendrocnide excelsa* Excelsatoxin A [31]
Cholesterol	Bacterial	*Streptococcus intermedius* intermedilysin [30]
		*Staphylococcus pyogenes* SLO [54]
	Mycotoxin	*Amanita phalloides* α-amanitin [36]
	Animal venom	*Chironex fleckeri* venom [41]
Sphingolipids	Bacterial	*Clostridium difficile* toxin A [55]
		*Escherichia coli* α-haemolysin [29]
		*Escherichia coli* Shiga toxin [26,35,101–103]
		*Staphylococcus aureus* Panton-Valentine leukocidin [52,53]
		*Staphylococcus pyogenes* SLO [54]
		*Vibrio cholerae* (‘Cholera’) toxin [73]
	Mycotoxin	*Amanita phalloides* α-amanitin [36]
	Animal venom/toxin	*Chironex fleckeri* venom [41]
		*Hadronyche infensa* gomesin [43]
Intracellular Process		
Retrograde transport	Bacterial	*Vibrio cholerae* (‘Cholera’) toxin [73]
		*Clostridium difficile* toxin A [55]
		*Escherichia coli* α-haemolysin [27]
		*Morganella morganii* Tc toxin [67]
		*Photorhabdus luminescens* Tc toxin [67]
		*Salmonella enterica* typhoid toxin [104]
	Plant	*Ricinus communis* ricin [35,73,104]
	Animal venom	*Chironex fleckeri* venom [41]
ER-associated degradation	Bacterial	*Salmonella enterica* typhoid toxin [104]
		*Vibrio cholerae* (‘Cholera’) toxin [73]
	Plant	*Ricinus communis* ricin [35]
Ubiquitination	Bacterial	*Bacillus anthracis* anthrax lethal toxin [105]
	Plant	*Dendrocnide excelsa* Excelsatoxin A [31]
	Mycotoxin	*Aspergillus spp.* AFB1 [38]
Mitochondrial function	Bacterial	Mitochondrial inhibitors (piericidin, antimycin, oligomycin, chloramphenicol) [106]
		*Streptomyces spp.* valinomycin [107]
	Mycotoxin	*Aspergillus spp.* AFB1 [38,80]
		*Fusarium spp.* fusaric acid [40]

Beyond cellular entry factors, common toxin-targeted intracellular processes include retrograde transport, ER-associated degradation, ubiquitination, and mitochondrial function. All of these processes are essential to healthy cellular function: retrograde transport machinery is required for shuttling biomolecules from the cell surface to the Golgi and ER for signalling and protein recycling, ER-associated degradation and ubiquitination remove misfolded or damaged proteins from the cells so as to prevent toxic accumulation and recycle amino acids, and mitochondrial function is required for producing energy necessary for the broad range of essential cellular functions. Just as toxins are targeting essential and ubiquitous components of the cell membrane for entry, so too are they hijacking critical endogenous processes to disturb cellular homeostasis and/or induce death in a broad range of cell types and organisms. Though alternative mechanisms of toxin-induced death have been observed, such as genotoxicity induced by formation of AFB1–DNA adducts, these mechanisms have not yet been identified as broadly employed by toxins of diverse origins.

It is worth noting that there likely exists overlap between these categories (i.e. both GAGs and sphingolipids may be N-glycosylated). Additionally, the identification of conserved targets by this review is not exhaustive. There is considerable variation in the choice of screening parameters (detailed in section ‘Limitations of CRISPR screening for toxin activity’) and many authors choose to validate or otherwise focus on only a subset of the genes and pathways implicated by their screen. Meta-analysis of screens with calling of hits based on a single analysis pipeline and with the same defined cut-offs for significance and change in read count abundance would provide a more comprehensive analysis and could serve as a valuable resource for future studies. Furthermore, as more toxin CRISPR screens are performed it is likely that additional conserved cellular entry factors and intracellular processes will be identified.

The predominant use of CRISPRko with cell death-based screening (with only a small number of toxin screens that identify sensitising or synthetic sick/lethal KOs) suggests that inhibition of implicated conserved cell entry or intracellular processes is protective against toxin action. In order to identify conserved mechanisms that are activating for toxin action, there is a need for (1) altered screen design, such as the use of lower toxin doses so that both sensitising and resisting genetic perturbations can be identified, and (2) use of CRISPRa and inhibition for dissecting underlying gene regulatory networks.

CRISPR screening functions as a powerful, unbiased method of rapidly interrogating the entire host cell genome for factors mediating toxin entry and activity and has demonstrated incredible utility in identifying previously uncharacterised mechanisms of intoxication. However, the convergence of toxin activities as discussed above suggests that a more rapid, less laborious and cheaper ascription of toxin function could be performed through preliminary pharmacologic targeting of conserved functions. For example, a small panel of compounds such as PNGase F, heparin, and/or xyloside derivatives, GPI-cleaving enzymes, cyclodextrins, cyclodextrin–cholesterol complexes, and sphingomyelinase could be used when treating cells with toxin and would indicate the role of N-glycans, GAGs, GPI anchors, cholesterol or sphingolipids, respectively, as mediating toxin cellular entry. In this way, small panels of drugs could be used to rapidly stratify novel toxins or venoms based on core cellular entry or intracellular targets for a toxin of interest (Table 3). CRISPR screening could then later be used if a complete molecular dissection of toxin mechanism of action is desired or unique cellular receptors and/or processes are suspected.

**Table 3:** Potential pharmacological modulators of conserved cell surface factor and intracellular processes targeted by toxins

Cellular target	Compound	Effect
Cellular entry		
N-glycans	PNGase F	Cleaves all N-glycans on cell surface
	Tunicamycin	Inhibits N-glycosylation through competitive inhibition of substrate UDP-GlcNAc [108]
GAGs	Heparin or heparinoids (i.e. tinzaparin, dalteparin)	Pre-treatment or co-treatment can bind exogenous toxins, inhibiting interactions with cell surface factors [42]
	Xyloside derivatives	Competitive inhibition of xylosylation step which initiates GAG biosynthesis [109]
	Heparanase	Selective degradation of heparan sulfate [110]
	Chondroitinase ABC	Selective degradation of both chondroitin sulfate and dermatan sulfate
	Hyaluronidase	Selective degradation of hyaluronic acid
GPI anchors	GPI-cleaving enzymes (e.g. phosphatidylinositol-specific phospholipase C, GPI-specific phospholipase D)	Cleave GPI anchors, freeing them from the cell surface
Cholesterol	Cyclodextrins (i.e. MβCD, HPβCD)	Deplete plasma membrane cholesterol
	Cholesterol–cyclodextrin complexes	Provide exogenous cholesterol for increasing plasma membrane cholesterol abundance
Sphingolipids	Myriocin	Blocks *de novo* sphingolipid synthesis by inhibiting serine palmitoyltransferase
	Sphingomyelinase D	Degrades plasma membrane sphingomyelin
Intracellular processes		
Retrograde transport	Retro-2	Inhibit transport from early endosomes to the trans-Golgi network [111]
ER-associated degradation	Kifunensine	Inhibits ER mannosidase I to prevent mannose degradation on precursor glycoproteins
Ubiquitination	TAK-243, Deubiquitinases	TAK-243 binds free ubiquitin, inhibiting the activation of ubiquitin-activating enzymes Deubiquitinases remove ligated ubiquitin, preventing targeted protein degradation
Mitochondrial function	Polyphenols, carotenoids (e.g. astaxanthin)	Polyphenols are antioxidants that deplete reactive oxygen species Carotenoids can quench singlet oxygen and scavenge oxygen radicals

## Limitations of CRISPR screening for toxin activity

Despite their utility, CRISPR screens used in characterising toxin activity are limited in several ways. Most notably, the vast majority of CRISPR screens have been KO screens. Furthermore, a limitation of CRISPRko screening is the toxicity of DSBs [112–114], which may result in genes in amplified genomic regions being called as false negatives when considering KOs conferring protection against intoxication [115,116]. Broader application of CRISPRa (or parallel screening with both CRISPRa and CRISPRi) may permit identification of novel receptors or host cell factors missed by CRISPRko screening, being able to increase or decrease gene expression rather than simply abolishing it. CRISPRa and CRISPRi permit targeting of non-protein coding genomic elements, expanding the searchable genotypic space to include gene regulatory regions, microRNAs and long non-coding RNAs, enable interrogation of gene function without DSB-induced genotoxicity, and through modulating gene regulatory networks allow for the building of systematic genetic interaction maps. It has also been suggested that CRISPRi and CRISPRa have fewer off-targets than CRISPRko owing to the narrow windows of sequence proximal to the transcriptional start site to which they must bind to be effective [73]. Additionally, CRISPRa can activate inactive genes or pathways within a cell, which may otherwise be inaccessible in CRISPRko or CRISPRi screens where the gene is not endogenously expressed.

To date, all screens conducted have also been characterised by single gene perturbation, which may result in missing synergistic effects of multiple genes or functional redundancies in pathways. For example, in a CRISPRko screen for α-amanitin cytotoxicity, STT3B KO was identified as a hit that inhibited cell death but subsequent combined KO of both STT3A and STT3B, members of the same complex, resulted in “near complete” protection [36]. Future screens could make use of combinatorial CRISPR screening strategies, such as the use of Cas12a-based enzymes that use multiple sgRNA to enable multiplexed gene perturbations [117–119]. Additionally, it is of critical importance that the effect of hit gene perturbation be validated in multiple cell lines owing to variation in endogenous gene expression and propensity for gene editing. For example, three separate studies performed CRISPR screens for AFB1 in different cell lines and each identified unique regulators of AFB1-mediated cytotoxicity [37,38,80].

Comparing toxin screens also highlights three common scenarios characterised by inconsistency in the identification of hit genes. Firstly, screens for different toxins that target the same cellular entry factor, process or pathway produce non-overlapping sets of gene hits. In this instance, each individual screen may hit some but not all genes associated with the shared entry factor / process / pathway. While this may be a result of distinct toxins interacting with a given pathway in different ways, non-overlapping gene hit lists can be found even when comparing screens that target the same surface receptor and so should identify the genes constituting its biosynthetic pathway, such as screens performed for Shiga toxin that has a well-characterised interaction with the Gb3 ganglioside [26,35,101–103,120]. Secondly, there are several instances of independent CRISPR screening studies being performed to characterise a single toxin but yielding contradictory findings, as evidenced by screens for *Ricinus communis* ricin and *Aspergillus* spp. AFB1 [34,35,37,38,73,80]. Thirdly, there are a number of studies that do not correlate toxin function with a known pathway, instead having a small number of genes with no known functional association (i.e. screens for *Bacillus cereus* Hemolysin BL and *Bordetella pertussis* dermonecrotic toxin [49,97]). The inconsistencies identified in all of these situations may be attributed to choices pertaining to critical design parameters when selecting a screening strategy. These include choice of: (1) cell line, which may yield varied results owing to cell type- and species-specific toxin mechanisms; (2) the selective pressure conferred by the concentration of toxin used, duration of toxin exposure and how many rounds of selection; (3) phenotypic selection step, which has most typically been toxin-induced death but also includes FACS- and MACS-based selection; (4) sgRNA library and the ruleset used to select individual sgRNA; and (5) the analysis pipeline used, including the software package and cut-offs for gene effect (i.e. LFC) and significance.

Of particular note are the latter of these design choices, namely sgRNA library choice and analysis pipeline. The majority of published toxin screens use commercially available whole-genome CRISPRko libraries, such as the GeCKOv2, TKOv3, and Brunello libraries. Each of these libraries are designed using different rule sets to predict sgRNA performance, often balancing on-target performance with chance for off-target mutagenesis, and so are composed of different sgRNAs. Given that sgRNA activity is dependent on variables such as chromatin accessibility and endogenous gene expression (which contribute to cell type-specific variability in sgRNA performance), position within a gene, GC content and both number and presence of nucleotides at particular positions [121–126], these libraries will have varied efficiency in targeting genes. In addition, the analysis pipeline used varies considerably between studies. Commonly used analysis software packages include MAGeCK, BAGEL, and CERES, though bespoke approaches see considerable adoption as well, such as ranking of hits by read count and number of sgRNA per gene. Best practice for the field would be to design screens that: feature a phenotypic selection strategy with multiple rounds of selection and collection of samples following each round for ranking of gene hits; are conducted in multiple cell lines to identify lineage-specific and lineage independent hits; avoid genetic bottlenecking; feature stringent significance cut-offs in statistical analysis; utilise validated sgRNA libraries; and which are followed by rigorous application of assays for hit gene validation.

Furthermore, most CRISPR screens have focused on toxin-induced death. There are a range of alternate readouts that may be used for phenotypic screening of toxin activity. One such readout is ligand–receptor interaction screening [127–131], which involves the labelling of ligands with fluorophores or magnetic particles so they can function as ‘bait’ for unknown receptors. This technique has already seen use in toxin screening, being used to study lectin binding [33]. Bioorthogonal labelling of biomolecules via click chemistry represents a nascent screening strategy, having been used to investigate O-GalNacylation of glycans [132] and phosphatidylcholine metabolism [133], and may also serve as a powerful tool for investigating cellular processes mediating intoxication (for example, labelling of glycans or their precursors to understand their role in toxin binding and function). Screening with biosensors such as dyes or genetically encoded indicators has been used to investigate a range of processes including lysosome homeostasis [134], cell division [135] and autophagosomal function [136,137] as well as GPCR/cAMP [138] and NF-kB signalling [139]. Choice of an appropriate biosensor may permit dissection of the involvement of such cellular processes in intoxication. Notably, a recent preprint reports use of the genetically encoded Ca^2+^ reporter CaMPARI2 for Ca^2+^-based screening in neuronal cells [140]. Such reporters may be useful in observing cationic flux associated with painful toxins and venoms. Pooled optical screening has also been used to observe changes in cellular morphology or behaviour via high content, time-resolved microscopy [141–143].

Given that the actions of toxins are complex, having various systemic and multi-organ effects, and noting that intoxication may involve the concerted action of multiple toxins, more complete characterisations of host cell factors mediating intoxication will necessitate the use of more complex phenotypic readouts, such as single cell-based screening (i.e. using RNA-seq, ATAC-seq, protein-level or spatial readouts) and screening *in vivo* to observe systemic effects of intoxication. In recent years, there has been a trend towards the development of highly parameterised and multi-modal screen designs, enabled by new methods in single-cell and spatial CRISPR screening. Notable examples include Perturb-map [144], Perturb-FISH [145], Perturb-multimodal [146], and spatial Perturb-seq [147]. These approaches combine CRISPR mutagenesis with optical imaging to enable identification of sgRNAs and through recording the spatial distribution and organisation of cells and tissues, have the distinct advantage of being able to dissect cell autonomous and non-autonomous behaviours. Multi-omic information may be gained by combining these spatial techniques with single cell sequencing for transcriptomic profiling or additional antibody-based stains for examining protein-level changes.

Notably, the aforementioned technologies have been used *in vivo*. Given that most screens are conducted *in vitro*, systemic effects of intoxication and interactions with the circulatory and immune systems of host organisms, which are critical factors in the symptomology and severity of intoxication events, are lost. Use of *in vivo* screens in the future would permit observation of the effects of genetic perturbations in whole organism intoxication. For example, just as Perturb-map was used to investigate perturbation-specific gene signatures and immune cell infiltration within tumours, so too could the spatial relationship of gene perturbations and tissue organisation be investigated in tissue sections of organs targeted by specific toxins or envenomed tissues. However, effective design of *in vivo* CRISPR screens and screening logic for these types of studies remains to be established.

## Conclusion

In summary, CRISPR screens have served to identify many unique and conserved mechanisms of intoxication. The identification of conserved mechanisms of cellular entry and targeting of intracellular processes across toxins derived from unrelated kingdoms suggests that panels of pharmacologic modulators or genetic controls may be used for crude first-pass screening of inhibitors of intoxication by a toxin of interest *in vitro* or as broad acting toxin antidotes targeting core pathways essential for toxin action. Importantly, use of CRISPR screening permits identification of novel cellular targets that would be missed by these targeted investigations and so remains a valuable tool for rigorous and unbiased dissection of toxin action. Additionally, for more holistic characterisation of systemic toxin effects, as well as the characterisation of intoxication with several toxins simultaneously (as is the case with envenomation), *in vivo* and multi-parameter screens will be required.

## Perspectives

- Toxins demonstrate incredible functional diversity. Characterising this functionality has informed treatment strategies for intoxication/envenomation events, permitted production of novel therapeutics, led to development of new tools for molecular biology research, and informed agricultural practices. However, the mechanism of action of many toxins is poorly or incompletely understood. Pooled CRISPR screens have been applied to elucidate the mechanism of toxin activities.
- The use of pooled CRISPR screens in characterising the activity of bacterial, plant, fungal and animal venoms/toxins highlights shared cell entry factors and targeting of specific intracellular processes. Conserved cell entry targets include N-glycans, GAGs, GPI anchors, cholesterol and sphingolipids, while conserved targeted intracellular processes include retrograde transport, ER-associated degradation, ubiquitination, and mitochondrial function.
- When investigating toxins of unknown function, small panels of pharmacologic modulators or genetic controls could be used to generally stratify toxin–host interactions on a first pass, with CRISPR screens being used for subsequent mapping of genome-wide host factors for toxins with unknown mechanisms. However, more thorough physiological characterisation of toxin activity, particularly systemic effects, may require screening *in vivo* and/or with more complex phenotypic readouts.

## Supplementary Material

Supplementary Table S1

## Open Access

Open access for this article was enabled by the participation of the University of Sydney in an all-inclusive Read & Publish agreement with Portland Press and the Biochemical Society under an agreement with CAUL.

## Abbreviations

αHL: α-hemolysin
AFB1: Aflatoxin B1
ATAC-seq: Assay for Transposase-Accessible Chromatin using sequencing
cAMP: cyclic adenosine monophosphate
CRISPR: Clustered Regularly Interspaced Short Palindromic Repeats
CRISPRa: CRISPR activation
CRISPRi: CRISPR interference
CRISPRko: CRISPR knockout
DSB: Double-stranded break
ER: Endoplasmic reticulum
ExTxA: Excelsatoxin A
FACS: Fluorescence-activated cell sorting
GAG: Glycosaminoglycan
GPCR: G-protein-coupled receptor
GPI: Glycosylphosphatidylinositol
ICG: Indocyanine green
KO: Knockout
LFC: Log-fold change
LPS: Lipopolysaccharide
MACS: Magnetic-activated cell sorting
NGS: Next-generation sequencing
PCR: Polymerase chain reaction
PFT: Pore-forming toxin
PVL: Panton-Valentine leukocidin
RNA: Ribonucleic acid
sgRNA: Single guide RNA
SLO: Streptolysin O
