# Supplementary material for: Dissecting conserved molecular mechanisms of biological toxin activity through CRISPR screening
Source: Biochem Soc Trans. 2026 May 28;54(6):601–20. doi: 10.1042/BST20250099 (PMC13223377; doi:10.1042/BST20250099)
Supplement: Supplementary Table S1 [file BST-2025-0099C_supp.pdf]

Supplementary Table 1: Toxin CRISPR screens considered in this literature review.
For bacterial toxins only, a recent representative publication is detailed for each unique toxin. Other published CRISPR screens for that toxin are included as additional references under the 'toxin' column.
* indicates a pathway or process inferred from gene hits.

Toxin	CRISPR Modality	Cell Line	Library	Selection	Notable Hit Genes	Notable Pathways or Processes
**Bacterial toxins**						
Aeromonas hydrophila proaerolysin (1)	CRISPRko	S2R+ (Fruit fly)	Custom	2 nM proaerolysin for 3 weeks	*CG46311*<br>GPI genes (e.g. *PIG-A, GAA1, PIG-K*, etc)<br>N-glycosylation genes (e.g., *Stt3B, nst, Mgat1*, etc)	GPI anchor synthesis, N-glycosylation
Bacillus anthracis anthrax lethal toxin (2)<br>Additional ref(s): (3, 4)	CRISPRko	$TLR4^{-/-}$ iBMDMs (Mouse)	Genome-wide Mouse Lentiviral CRISPR gRNA library v1 (5)	1 µg/mL anthrax lethal toxin for 5 hours	*Antxr2, Casp1, Nlrp1b, Ubr2*	Pyroptosis, ubiquitination
Bacillus cereus Hemolysin BL (HBL) (6)	CRISPRko	(1) RAW276.4 (Mouse)<br>(2) HT1080 (Human)<br>(3) B16F10 LITAF-KO cells (Mouse)	(1) Mouse GeCKOv2 library A (7)<br>(2) GeCKOv2	2 rounds of selection with 2.5 nM HBL for 2 h followed by recovery in toxin-free media for 2 days	(1 and 2) *LITAF*<br>(3) *CDIP1*	None identified
Bordetella pertussis dermonecrotic toxin (8)	CRISPRko	MC3T3-E1 (Mouse)	Genome-wide Mouse Lentiviral CRISPR gRNA library v1	3 rounds of selection with 2 µg/mL DNT-DT_A for 36 h followed by three washes and reseeding in toxin-free media	*Cacna1g, Taok1, Dhx29, Nrg2, Tmem151b*	None identified
Clostridium difficile toxin A (9)	CRISPRko	HeLa (Human)	GeCKOv2 (7)	(1) 3 rounds of selection with increasing concentrations (40, 80 and 160 pM) of truncated *C. difficile* toxin A (TcdA) for 48 h then cultured in toxin-free media until ~70% confluent<br>(2) Screen using full length TcdA	Truncated TcdA screen: *LDLR, EXT2, EXRL3, HS6ST1, NDST1, SLC35B2, UGP2, PI4KB, ATP6V0D1, COG5, COG7, TMEM165, RIC8A*<br>Full length TcdA screen: *UGP2, SGMS1, ZNF283*	Heparan sulfate biosynthesis and sulfation, Golgi apparatus and glycosylation, glycosaminoglycan synthesis, sphingolipid biosynthesis, lipid raft formation and endocytosis
Clostridium novyi alpha-toxin (Tcnα) (10)	CRISPRko	HeLa (Human)	GeCKOv2	3 rounds of selection with Tcnα	*B4GALT7, B3GALT6, EXT1, SLC35B2, B3GAT3, EXT2, HS6ST3, EXTL3*	Heparan sulfate biosynthesis
Clostridium septicum alpha-toxin (5)	CRISPRko	JM8-Cas9 embryonic stem cells (Mouse)	Custom<br>(1) Targeting 26 GPI-anchor	1 nM alpha-toxin for 48 h, then cultured for another 5 days	*B4galt7, 1700016K19Rik, Cstf3, Ext2*, known GPI pathway genes (i.e., *Pgap2, Piga, Pigl, Pigm*)	GPI anchor biosynthesis, mismatch repair

Toxin	CRISPR Modality	Cell Line	Library	Selection	Notable Hit Genes	Notable Pathways or Processes
			biosynthesis pathway genes (2) Whole genome		and mismatch repair genes (i.e., *Mlh1, Msh2, Msh6, Pms2*)	
Clostroidies difficile toxin B (11)<br>Additional ref(s): (12-14)	CRISPRko	HeLa<br>(Human)	GeCKOv2	3 rounds of selection with type 4 toxin B (sequence variant 2) supernatant with increasing concentrations (1/500 000, 1/250 000, and 1/125 000 v/v) for three days	*TFPI, PIGS, PIGV, PIGM, PIGU, GPAA1, UGP2, PIGB, PIGF, PIGX, PIGY, PIGA*	GPI anchor biosynthesis
Corynebacterium diphtheriae (‘Diphtheria’) toxin (15)<br>Additional ref(s): (4, 16, 17)	CRISPRko	PK-15<br>(Porcine)	Custom	2 rounds of selection with 2 ng mL^{-1} diphtheria toxin for 10 days	*HBEGF, DPH1, DPH2, DNAJC24, ZBTB17*	Diphthamide biosynthesis
Enterococcus pore-forming toxin 2 (Epx2) (18)	CRISPRko	HeLa<br>(Human)	GeCKOv2	Selection at 0.25 µg/mL Epx2 for 24 h, then cultured in toxin-free media until 70% confluent, then selected at 0.5 µg/mL for 24 h.	*B2M, SNX17, GAGE1, HLA-A*	None identified
Escherichia coli Shiga toxin (19)<br>Additional ref(s): (20-24)	CRISPRko	*SPTLC1* KO HeLa<br>(Human)	GeCKOv2	Cells treated with 2 µM sphingosine-1-phosphate. The following day, cells selected with 2.5 ng/mL Shiga toxin for 15 days	*A4GALT, B4GALT5, UGCG, CERS, LAPTM4A, TM9SF2, SLC35A2, TMEM165, GOLPH3, AHR, SGPP1, PLPP3*	Sphingolipid synthesis, Gb3 synthesis
Escherichia coli subtilase cytotoxin (SubAB) (25)	CRISPRko	HeLa<br>(Human)	GeCKOv2	7.5 ng/ml SubAB for 24 h, left to recover for 11 days, then treated for further 24 h with 10 ng/ml	*SLC35A1, MGAT1, UNC50, KDELR2, VPS53, COG4, CAMLG, COG3, SLC35A2, VPS54, COG6, COG5, WRB, PTAR1, VPS51, GOSR1, SLC39A9, ASNA1, CMAS, GET4, ARL1, C1GALT1, COG2, JTB, COG1, GOSR2, COG7, KDELR1, VPS52, C1GALT1C1, COG8, NAPG, KIAA1432*	Sialglycans (N-glycans, O-glycans), membrane trafficking
Escherichia coli α-haemolysin (26)	CRISPRko	IMCD-3<br>(Mouse)	Brie	3 rounds of selection with 10 hemolytic units of α-haemolysin for 2 h (~ 60% cell death) then recovery for 48 h	*Aagab, Ap2s1, Ap2m1, Armh3m Cltc, Cyfip1, Ldlr, Nckap1, Npc1, Pi4kb, Smdt1, Vps11*	Clathrin-mediated endocytosis, vesicle transport
Lipopolysaccharide (LPS) (27)<br>Additional ref(s): (28-33)	CRISPRko	THP-1 macrophages<br>(Human)	Custom sub-genomic (74 gRNAs for 35 target genes)	Cells treated with 100 ng/mL LPS for 3 hours and then fixed before undergoing procedure for spatial transcriptomics and	*MAP3K7, IRAK1, TRAF6, RELA, MYD88*	NF-κB pathway

Toxin	CRISPR Modality	Cell Line	Library	Selection	Notable Hit Genes	Notable Pathways or Processes
				optical *in situ* sgRNA detection ('Perturb-FISH')		
Micromonospora purpurea gentamycin (34)	CRISPRi	*Escherichia coli* K-12 MG1655	Custom	Cells seeded at optical density at 600 nm (OD_{600}) of 0.05, then cultured in 1 ug/mL gentamycin until OD_{600} of 1.68	Ribosome and ribosome-associated proteins, toxin-antitoxin system genes, outer membrane proteins and efflux pumps	Coenzyme transport and metabolism, riboflavin biosynthesis, quinone biosynthesis, heme biosynthesis
Mitochondrial inhibitors (antimycin, chloramphenicol, oligomycin, piericidin) (35)	CRISPRko	K562<br>(Human)	Brunello (36)	100 nM antimycin, 10 μg/mL chloramphenicol, 10 nM oligomycin, 10 nM piericidin, or 10 nM antimycin + 10 nM oligomycin for 15 days	Synthetic sick/lethal: *GPX4, GOT1, G6PD, PFKP, GPI, ALDOA, HK2, PGP, RPE*<br>Epistatic buffering: *REXO2, LARP1*<br>Suppressors: *ATPIF1, vHL, TCEB2, NAIF1, NR2F2, SIN3A, SUDS3, KLF16, C7orf26, FAM122A, NDUFA9*	Mitochondrial function, glycolysis, pentose phosphate pathway
Morganella morganii Tc toxin (mTc) (37)	CRISPRko	S2R+<br>(Fruit fly)	Custom	3 rounds of selection with increasing concentrations of mTc (550 pM, 1100 pM, and 3600 pM) for 1 week, passed through 30 μM cell strainer and then cultured in toxin-free media until ultra-confluent before proceeding with next round. Cells collected for gDNA isolation after each round.	*sgl, VAC14, FIG4, Cp1, ena*	Sulfated glycosaminoglycans, PIKfyve complex
Paeniclostridium sordellii hemorrhagic toxin (TcsH) (38)	CRISPRko	HT-29<br>(Human)	GeCKOv2, TKOv3 {Hart, 2017 #2}	4 rounds of selection with increasing amounts of TcsH added for 12-18 h (20, 50, 100, and 200 pM)	GeCKOv2: *UGT1A9, GMDS, FUT4, TMPRSS2, GPC2, SLC35C1, CNOT1, FGF20*<br>TKOv3: *CASP2, GMDS, H2AFV, SLC35C1, FUT4, TMPRSS2, PLCB3, MLLT6*	Fucosylation of cell surface glycans
Paeniclostridium sordelli lethal toxin (TcsL) (39)<br>Additional ref(s): (40)	CRISPRko	Hap1<br>(Human)	TKOv3	TcsL added at either 0.1 nM or 1 nM for 48 h	0.1 nM: *SEMA6A, UGP2, MAU2, DPM1, TBC1D21, ZNF592, NEK11*<br>1 nM: *SEMA6A, UGP2, DPM1, TAF4, ROR2, GSK3A*	Axon guidance, UDP sugar synthesis
Pasteurella multocida toxin (PMT) fusion protein (inactive PMT + active diphtheria toxin (PMT-DTa)) (16)	CRISPRko	Mouse embryonic fibroblast<br>(Mouse)	Mouse Improved Genome-wide Knockout CRISPR library v2 (41)	3 rounds of selection with 2 μg/mL PMT-DTa for 36 h, then 24 h in toxin-free medium	*Lrp1, Dph1, Gsdmcl1, Mical2, Gse1, Aatk, Lrrc16b, Trim66, Dph5, Clip1*	Endocytosis

Toxin	CRISPR Modality	Cell Line	Library	Selection	Notable Hit Genes	Notable Pathways or Processes
Photorhabdus luminescens Tc toxin (42) Additional ref(s): (37)	CRISPRko	HeLa (Human)	GeCKOv2	3 rounds of selection with *Photorhabdus luminescens* W14 Tc toxin (at increasing concentrations of 5, 10 and 20 nM) for 24 h	*MGAT2, MGAT1, MAN1A2, ACTG1*	N-glycan synthesis, sulfated glycosaminoglycans
Salmonella enterica typhoid toxin (43)	CRISPRko	HEK293T (Human)	GeCKOv2	40 pM of typhoid toxin for 60 min and then cultured in toxin-free media for 15 days	Lib A: *UNC50, COG6, TMEM165, ARL1, SYVN1, VPS54, SLC35A1, VPS51, C1GAL1, SLC35A2, YKT6, VPS52, COG1, COG5, POMGNT2, NMT1* Lib B: *TMEM165, VPS54, SLC35A1, ARL1, COG5, SEL1L, UNC50, SYVN1, VPS51, TMED2, SLC35A2, COG1, YIPF5, COG4, COG7, COG8, COPB1, GPR107, COPB2, TM9SF2, SCYL1*	Protein transport, glycoprotein metabolic process, glycosylation, Golgi organisation, ER to Golgi vesicle-mediated transport, retrograde transport endosome to Golgi, retrograde vesicle-mediated transport Golgi to ER, Intra-Golgi vesicle-mediated transport
Staphylococcus aureus Panton-Valentine leukocidin (PVL) (44) Additional ref(s): (45)	CRISPRko	THP1 macrophages (Human)	Human CRISPR Knockout Pooled Library (Brunello) (36)	PVL at 2 µg/ml for 24 h, viable cells sorted and collected by flow cytometry	*FBXO11*	Lipid biosynthesis, gene expression, post-translational modification, sphingolipids*
Staphylococcus aureus α-hemolysin (46)	CRISPRko	U937 (Human)	GeCKOv2	0.5 µg/mL α-haemolysin for 7 days	*ADAM10, SYS1, ARFRP1, TSPAN14, SGMS1*	Sphingomyelin synthesis, intracellular trafficking
Streptococcus agalactiae β-hemolysin/cytolysin (47)	CRISPRko	HeLa (Human)	GeCKOv2	3 rounds of 2 h incubation (3 lytic units), ~2 weeks recovery on puromycin media in between	*PLA2G15, VPS13A* (NB: Did not validate)	Glycerophospholipids
Streptococcus intermedius intermedilysin (48) Additional ref(s): (47)	CRISPRko	HAP1 (Human)	TKOv3	2 ng/mL intermedilysin for 1 h, two weeks for recovery, then 10 ng/mL intermedilysin for 1 h	*CD59, SREBF2, LDLR, MOGS, PRKCSH, GANAB, MGAT1, SSR1, SSR2, SSR3, UGP2, GALE, UXS1, XYLT2, NDST1, EXT1, EXT2, B3GALT6, B4GALT7, UGCG, SLC35A2, TM9SF2*	GPI-anchor synthesis and attachment, nucleotide sugar synthesis, lipid and protein glycosylation; heparan sulfate pathway, cholesterol metabolism pathway, N-glycosylation, UDP-sugar synthesis, ganglioside synthesis
Streptococcus pyogenes Streptolysin O (SLO) (49)	CRISPRko	HAP1 (Human)	GeCKOv2	Native SLO: 5 rounds of selection starting at 5 nM and increasing 2-fold each round. Cells incubated for 24 h	Native SLO: *UGCG, SREBF2, LDLR, B4GALT5* SLO L562D: *SREBF2, GALE, LDLR, UGCG*	Cholesterol biosynthesis, Glycosphingolipid biosynthesis

Toxin	CRISPR Modality	Cell Line	Library	Selection	Notable Hit Genes	Notable Pathways or Processes
				SLO L562D: 4 rounds of selection starting at 1 μM and increasing 2-fold each round. Cells incubated for 24 h		
Streptomyces spp. Valinomycin (50)	CRISPRko	YFP-Parkin MEFs (Mouse)	Mouse GeCKOv2	3 rounds of treatment with either 300 or 600 nM of Valinomycin	*Pink1*	Mitophagy
Vibrio cholerae ('Cholera') toxin / diphtheria toxin catalytic A subunit fusion protein (CTx-DTA) (17)	(1) CRISPRi (dCas9 or dCas9-KRAB)<br>(2) CRISPRa (sunCas9)	K562<br>(Human)	Custom	2 rounds of selection with 0.4 nM CTx-DTA for 24 h over 10 days.	CRISPRi: *SYVN1, SEL1L, UBXN4, SEL1L, B4GALNT1, CANX, UBEA4, C7ORF26*<br>CRISPRa*: GAL3ST1*	Infection with *Vibrio cholerae*, glycosphingolipid biosynthesis, ganglio-series, ribosome, proteasome, diphthamide biosynthetic pathway, ganglioside biosynthetic pathway, ER-associated degradation (ERAD), glycosphingolipid biosynthesis Lacto/Neolacto-series
**Plant toxins (Phytotoxins)**						
Dendrocnide excelsa Excelsatoxin A (ExTxA) (51)	CRISPRko	TE-671<br>(Human)	TKOv3	3 rounds of co-incubation with 1 μM ExTxA, 5 μM veratridine and 20 nM ouabain for 72 h	Protective:<br>*SCN9A, TMEM233, RNF121, GPAA1, PIGT, CRELD1, PIGK, STT3B, PIGS, MMGT1, LMAN2L*<br>Sensitising:<br>*SPAG5, TMEM161B, NEDD4L*	Ubiquitination, N-glycosylation*, GPI anchor synthesis*
Hippeastrum hybrid lectin (HHL) (52)	(1) CRISPRko<br>(2) CRISPRi (KRAB-dCas9)	A549<br>(Human)	(1) Custom KO library (Morgens et al., 2017)<br>(2) Custom CRISPRi sublibrary (292 genes, based on KO screen hits and related genes)	(1) Knockout screen: Doxycycline treatment to induce XBP1s for 48 h, then fixed and stained with FITC-labelled HHL. Sorted top and bottom 25% of HHL signal<br>(2) CRISPRi screen: Cells either dox-treated or untreated for 48 h. Cell suspension then incubated with HHL-coupled magnetic beads and three rounds of magnetic separation performed	**(1) CRISPRko screen:**<br>*MAN2A1, MGAT1, SLC35A2, WRB, MAN1A2, RMC1, ASNA1, TM9SF3, RAB7*<br>**(2) CRISPRi screen:**<br>Increased binding: *MAN1A1, MAN1A2, MGAT1, COG1-8, GET1, TM9SF3*<br>Decreased binding:<br>*CCDC22, CCDC93, VPS35L, LEO1, SMG7*	N-glycosylation
Lignocellulose hydrolysate toxins (i.e., aliphatic acids, furan aldehydes,	CRISPRi (dCas9-Mxi1) with anhydrotetracycline (ATx)-inducible sgRNA	BY4743 *Saccharomyces cerevisiae*<br>(Yeast)	Custom sub-genomic - 161 transcription factors and 129 protein kinases	Cells grown in synthetic complete media (SCM), SCM with 10% hydrolysate or SCM with 45% inhibitor cocktail for approx. 25 generation, all +/-	**Hydrolysate screen**<br>Protective: *DOT6, SKO1, BUB1*<br>Sensitizing: *UME6, CDC15, UGA3, PBS2, HOG1, HAA1, YAP1*<br>**Toxic inhibitor cocktail**	Oxidative, osmotic, acidic and general stress, HOG pathway signalling

Toxin	CRISPR Modality	Cell Line	Library	Selection	Notable Hit Genes	Notable Pathways or Processes
phenolic/aromatic derivatives) (53)			involved in regulation of growth adaptation and cellular signalling	ATc to induce sgRNA expression.	Protective: *BUB1*<br>Sensitizing: *STB5, YAP1, UME6*	
Maytenus spp. maytansine and maytansine antibody-drug conjugates (54)	CRISPRko	Ramos cells (Human)	Morgens et al. sgRNA library used for initial screen with maytansine ADC, then sub-genomic library targeting endolysosomal trafficking regulators, lysosome-localized genes and ~ 160 hits from previous screen used for free maytansine and maytansine ADC screens ('Targeted screen'). Later performed screen for sensitizing hits with library for ~ 2000 genes including drug targets, kinases, phosphatases	Targeted screen: Multiple rounds of selection with 0.2 nM maytansine or maytansine ADCs for 48 h<br>Sensitizing hit screen: 3 rounds of selection with 0.1 nM maytansine for 24 h	Targeted screen:<br>Protective: *MEN1, PGP, HK2*<br>Sensitising: *WDR81, RAB7A, BUB1*<br>Sensitising screen:<br>Protective: *HK2*<br>Sensitising: *BUB1*	None identified
Phaseolus vulgaris leucoagglutinin (PHA-L) (55)	CRISPRko	HL-60 (Human)	Human GlycoGene sgRNA library	2 rounds of FACS: 20 min incubation of cell suspension with 10 µg/mL fluorescent PHA-L	Inhibiting: *MGAT2, MGAT5, MGAT1*	N-glycan biosynthesis

Toxin	CRISPR Modality	Cell Line	Library	Selection	Notable Hit Genes	Notable Pathways or Processes
Ricinus communis ricin (17)	(1) CRISPRi (dCas9 or dCas9-KRAB)<br>(2) CRISPRa (sunCas9)	K562<br>(Human)	Tiling screens targeting the TSS of 49 known genes controlling ricin susceptibility	3 or 4 pulses of ~0.5 ng/mL ricin (LD50) over 16 days	Observed the expected phenotype for all 49 genes in CRISPRi screen; anticorrelated phenotype observed in subset of genes in CRISPRa screen	Retrograde trafficking
Ricinus communis ricin (56)	CRISPRko	K562<br>(Human)	Custom	4 rounds of selection with 0.25 ng/mL ricin toxin for 24 h then cells allowed to recover to normal doubling rate before next round	Protective: *DOLK, ALG14, ALG1, ALG2, ALG11, RFT1, ALG12, ALG5, ALG8, ALG10, ALG10B, OST4, MOQS, QANAB, MAN1B1, PMM2, GMPPS, GMDS, TSTA3, SLC35C1, SLC35A2, UGP2, GALE, MAN1A1, MGAT1, MAN2A1, MAN2A1, MGAT2, MGAT4B, MGAT5, FUTS, BUT4, B4GALT1*<br>Sensitizing*:*<br>*MGAT3, ST3GAL2*	Nucleotide sugar synthesis, N-glycan synthesis pathway
Ricinus communis ricin (23)	CRISPRko	HeLa<br>(Human)	GeCKOv2	4 rounds of selection with ricin (increasing concentrations of 0.2, 0.4, 0.8, and 1.5 ng/mL) for 48 h	*GOSR1, JTB, NBAS, TMEM165, TM9SF2, ALG5, ALG6, ALG8, MOGS, OST4, MAN1A2, MAN2A1, MGAT1, MGAT2, TSTA3, GMDS, SLC35C1, FUT4, VPS51, VPS51, VPS53, VPS54, GOSR1, NBAS, STX5, NAPG, ARL5B, ERP44, UBE2G2*	N-linked protein glycosylation, fucosylation, membrane trafficking, ER-associated protein degradation (ERAD)/quality-control pathways
Various chemotherapeutics (57)<br>Additional ref(s): (58-61)	CRISPRko	HAP1<br>(Human)	GeCKOv2	Cells selected with drugs for 14 days	*C1orf115 (RDD1), MGA, PPP6C, TOP1*	None identified
**Fungal toxins (mycotoxins)**						
Amanita phalloides α-amanitin (62)	CRISPRko	HAP1<br>(Human)	Brunello	1.5 μM α-amanitin (LD50) for 7 days	*TAF4, SLC46A3, MGAT1, SPPL3, WDR81, PSENEN, KIAA1033, STT3B, TIGD5, LAMTOR2*	Apoptosis, N-glycan biosynthesis, cholesterol metabolism, colorectal cancer, neurotrophin signalling pathway, necroptosis, sphingolipid signalling pathway
Aspergillus spp. aflatoxin B1 (63)	CRISPRko	PLC/PRF/5<br>(Human)	Brunello	6 rounds of 8 μM AFB1 for 48 h	*AHR, POR, KEAP1, SAFB, ALAS1, MYC, ONECUT1, WDR83, SLC5A5, RHEB*	Genotoxicity

Toxin	CRISPR Modality	Cell Line	Library	Selection	Notable Hit Genes	Notable Pathways or Processes
Aspergillus spp. aflatoxin B1 (64)	CRISPRko	PK-15 (Porcine)	PigGeCKO	3 rounds of AFB1 with increasing doses (0.2 µg/mL, 1 µg/mL, 6 µg/mL). Media refreshed daily until all control cells killed.	*TAF1C, OSTN, FOSL2, AFM, SMARCA4, BACH1, MFSD5, UBTD1, HOXA6, IZUMO2*	Oxidation-reduction processes
Aspergillus spp. aflatoxin B1 (65)	CRISPRko	IPI-2I (Porcine)	Custom	3 rounds of AFB1, Increasing concentration from 5 µg/mL to 8 µg/mL	*CBS, INHBA*	Mitochondrial protein methylation, impaired mitochondrial function, oxidative stress, ubiquitination
Candida albicans candidalysin (66)	CRISPRko	TR146 (Human)	Brunello	5 rounds of 30 µM candidalysin for 6h	*B3GALT6, TYK2, XYLT2, B3GAT3, SLC39A9, GBF1, EMP1*	GAG biosynthesis
Fusarium spp. fusaric acid (67)	CRISPRko	IPEC-J2 (Porcine)	Custom	50 µg/mL fusaric acid for at least 7 days (Until all control group cells died)	*TP53, MDH2, PDHB, G6PD, PDP1, CS, STOML2, LIAS, NDUFS4, USP28*	TCA cycle, pyruvate metabolism, carbon metabolism, glycolysis/gluconeogenesis
Fusarium spp. T-2 toxin (68)	CRISPRko	J774A.1 (Mouse)	Mouse GeCKO	25 nM T-2 toxin until nontarget control cells completely died	*Trp53inp1, St3gal3, RANbp6*	Pyroptosis, N-glycan biosynthesis*
**Animal toxins/venoms**						
Chironex fleckeri venom *(69)*	CRISPRko	HAP1 (Human)	GeCKOv2	1 µg/mL *C. fleckeri* venom for 14 days	*ATP2B1, MBTPS1, MBTPS2, SCAP, SGMS1, SREBF2*	Regulation of cholesterol biosynthesis by SREBP (SREBF), apoptotic cleavage of cell adhesion proteins, other semaphorin interactions, Endosomal Sorting Complex Required for Transport (ESCRT), amino acid synthesis and interconversion (transamination), metalloprotease DUBs, regulation of TP53 activity through phosphorylation, signalling by retinoic acid, sphingolipid *de novo* biosynthesis
Naja pallida venom *Naja nigricollis* venom *(70)*	CRISPRko	HAP1 (Human)	TKOv3	5 µg/mL *N. pallida* or *N. nigricollis* venom for 9 days	*Naja pallida*<br>Sensitizing: *SMARCD1, CDK13, HDAC3, ZFAT, CRAMP1L*<br>Protective: *TMEM50A, LEPROTL1, NDST1, XYLT2, EXT1, EXTL3, SLC35B2*<br>*Naja nigricollis*	*N. pallida:*<br>Heparan sulfate, GAG-protein linkage region, chondroitin sulfate, dermatan sulfate, heparan sulfate (late stages), aspartate degradation II, HIPPO signalling, PXR

Toxin	CRISPR Modality	Cell Line	Library	Selection	Notable Hit Genes	Notable Pathways or Processes
					Sensitising: *TSC1, TSC2, TBC1D7, SMARCC1, INPPL1, APPBP2*<br>Protective: *LEPROTL1, TMEM50A, EXT1, B4GALT7, EXT2, EXTL3, XYLT2, NDST1, SLC35B2*	signalling, L-cysteine degradation III, thyroid hormone metabolism II<br>*N. nigricollis:*<br>Heparan sulfate, GAG-protein linkage region, chondroitin sulfate, dermatan sulfate, heparan sulfate (late stages), pre-mRNA, L-glutamine biosynthesis II, thyroid hormone metabolism II, molybdenum cofactor, BCKDC
Hadronyche infensa gomesin (HiGom) (71)	CRISPRko	MM96L (Human)	TKOv3	4 rounds of 25 µM HiGom (LD90) for 72 h followed by 72 h in regular culture media	Protective: *CISD1, SAP30BP, CCL5, EDNRA, CYBRD1, TM4SF20, UGP2, CMAS, ST3GAL5, B4GALT5, UGCG*<br>Sensitising: *OPRL1, MIS18A, C4orf40*	CMP-N-acetylneuraminate biosynthesis I (Eukaryotes), GDP-mannose biosynthesis, sialic acid metabolism, colonic acid building blocks biosynthesis

References

1. Viswanatha R, Entwisle S, Hu Y, Kim A-R, Reap K, Butnaru M, et al. Higher resolution pooled genome-wide CRISPR knockout screening in Drosophila cells using integration and anti-CRISPR (IntAC). Nature Communications. 2025;16(1):6498.
2. Xu H, Shi J, Gao H, Liu Y, Yang Z, Shao F, et al. The N-end rule ubiquitin ligase UBR2 mediates NLRP1B inflammasome activation by anthrax lethal toxin. EMBO J. 2019;38(13):e101996.
3. Liu J, Zuo Z, Zou M, Finkel T, Liu S. Identification of the transcription factor Miz1 as an essential regulator of diphthamide biosynthesis using a CRISPR-mediated genome-wide screen. PLoS Genet. 2020;16(10):e1009068.
4. Zhou Y, Zhu S, Cai C, Yuan P, Li C, Huang Y, et al. High-throughput screening of a CRISPR/Cas9 library for functional genomics in human cells. Nature. 2014;509(7501):487-91.
5. Koike-Yusa H, Li Y, Tan EP, Velasco-Herrera Mdel C, Yusa K. Genome-wide recessive genetic screening in mammalian cells with a lentiviral CRISPR-guide RNA library. Nat Biotechnol. 2014;32(3):267-73.
6. Liu J, Zuo Z, Sastalla I, Liu C, Jang JY, Sekine Y, et al. Sequential CRISPR-Based Screens Identify LITAF and CDIP1 as the Bacillus cereus Hemolysin BL Toxin Host Receptors. Cell Host Microbe. 2020;28(3):402-10 e5.
7. Sanjana NE, Shalem O, Zhang F. Improved vectors and genome-wide libraries for CRISPR screening. Nat Methods. 2014;11(8):783-4.
8. Teruya S, Hiramatsu Y, Nakamura K, Fukui-Miyazaki A, Tsukamoto K, Shinoda N, et al. Bordetella Dermonecrotic Toxin Is a Neurotropic Virulence Factor That Uses Ca(V)3.1 as the Cell Surface Receptor. mBio. 2020;11(2).
9. Tao L, Tian S, Zhang J, Liu Z, Robinson-McCarthy L, Miyashita SI, et al. Sulfated glycosaminoglycans and low-density lipoprotein receptor contribute to Clostridium difficile toxin A entry into cells. Nat Microbiol. 2019;4(10):1760-9.
10. Zhou Y, Li D, Luo J, Chen A, Li X, Pan Z, et al. Sulfated glycosaminoglycans and low-density lipoprotein receptor mediate the cellular entry of Clostridium novyi alpha-toxin. Cell Res. 2021;31(8):935-8.
11. Tian S, Xiong X, Zeng J, Wang S, Tremblay BJ, Chen P, et al. Identification of TFPI as a receptor reveals recombination-driven receptor switching in Clostridioides difficile toxin B variants. Nat Commun. 2022;13(1):6786.
12. Guo S, Chen Y, Liu J, Zhang X, Liu Z, Zhou Z, et al. Low-density lipoprotein receptor-related protein 1 is a CROPs-associated receptor for Clostridioides infection toxin B. Sci China Life Sci. 2022;65(1):107-18.
13. Tao L, Zhang J, Meraner P, Tovaglieri A, Wu X, Gerhard R, et al. Frizzled proteins are colonic epithelial receptors for C. difficile toxin B. Nature. 2016;538(7625):350-5.
14. Luo J, Yang Q, Zhang X, Zhang Y, Wan L, Zhan X, et al. TFPI is a colonic crypt receptor for TcdB from hypervirulent clade 2 C. difficile. Cell. 2022;185(6):980-94 e15.
15. Yu C, Zhong H, Yang X, Li G, Wu Z, Yang H. Establishment of a pig CRISPR/Cas9 knockout library for functional gene screening in pig cells. Biotechnology Journal. 2022;17(7):2100408.

16. Schoellkopf J, Mueller T, Hippchen L, Mueller T, Reuten R, Backofen R, et al. Genome wide CRISPR screen for Pasteurella multocida toxin (PMT) binding proteins reveals LDL Receptor Related Protein 1 (LRP1) as crucial cellular receptor. PLoS Pathog. 2022;18(12):e1010781.
17. Gilbert LA, Horlbeck MA, Adamson B, Villalta JE, Chen Y, Whitehead EH, et al. Genome-Scale CRISPR-Mediated Control of Gene Repression and Activation. Cell. 2014;159(3):647-61.
18. Xiong X, Tian S, Yang P, Lebreton F, Bao H, Sheng K, et al. Emerging enterococcus pore-forming toxins with MHC/HLA-I as receptors. Cell. 2022;185(7):1157-71 e22.
19. Kono M, Hoachlander-Hobby LE, Majumder S, Schwartz R, Byrnes C, Zhu H, et al. Identification of two lipid phosphatases that regulate sphingosine-1-phosphate cellular uptake and recycling. J Lipid Res. 2022;63(6):100225.
20. Sakuma C, Sekizuka T, Kuroda M, Hanada K, Yamaji T. Identification of SYS1 as a Host Factor Required for Shiga Toxin-Mediated Cytotoxicity in Vero Cells. Int J Mol Sci. 2021;22(9).
21. Majumder S, Kono M, Lee YT, Byrnes C, Li C, Tuymetova G, et al. A genome-wide CRISPR/Cas9 screen reveals that the aryl hydrocarbon receptor stimulates sphingolipid levels. J Biol Chem. 2020;295(13):4341-9.
22. Yamaji T, Sekizuka T, Tachida Y, Sakuma C, Morimoto K, Kuroda M, et al. A CRISPR Screen Identifies LAPTM4A and TM9SF Proteins as Glycolipid-Regulating Factors. iScience. 2019;11:409-24.
23. Tian S, Muneeruddin K, Choi MY, Tao L, Bhuiyan RH, Ohmi Y, et al. Genome-wide CRISPR screens for Shiga toxins and ricin reveal Golgi proteins critical for glycosylation. PLoS Biol. 2018;16(11):e2006951.
24. Pacheco AR, Lazarus JE, Sit B, Schmieder S, Lencer WI, Blondel CJ, et al. CRISPR Screen Reveals that EHEC's T3SS and Shiga Toxin Rely on Shared Host Factors for Infection. mBio. 2018;9(3).
25. Yamaji T, Hanamatsu H, Sekizuka T, Kuroda M, Iwasaki N, Ohnishi M, et al. A CRISPR Screen Using Subtilase Cytotoxin Identifies SLC39A9 as a Glycan-Regulating Factor. iScience. 2019;15:407-20.
26. Kuhn HW, Smither MR, Jin RJ, Collins CA, Ma H, Sina J, et al. LDL receptor-mediated endocytosis of Escherichia coli alpha-hemolysin mediates renal epithelial toxicity. Proc Natl Acad Sci U S A. 2025;122(24):e2505482122.
27. Binan L, Jiang A, Danquah SA, Valakh V, Simonton B, Bezney J, et al. Simultaneous CRISPR screening and spatial transcriptomics reveal intracellular, intercellular, and functional transcriptional circuits. Cell. 2025;188(8):2141-58.e18.
28. Li Z, Feng Z, Chen M, Shi X, Cui B, Sun Y, et al. Rbfox3 Promotes Transformation of MDSC-Like Tumor Cells to Shape Immunosuppressive Microenvironment. Adv Sci (Weinh). 2025;12(8):e2404585.
29. Lampson BL, Ramiotarez AS, Baro M, He L, Hegde M, Koduri V, et al. Positive selection CRISPR screens reveal a druggable pocket in an oligosaccharyltransferase required for inflammatory signaling to NF-kappaB. Cell. 2024;187(9):2209-23 e16.
30. Tong J, Wang X, Liu Y, Ren X, Wang A, Chen Z, et al. Pooled CRISPR screening identifies m(6)A as a positive regulator of macrophage activation. Sci Adv. 2021;7(18).

31. Benaoudia S, Martin A, Puig Gamez M, Gay G, Lagrange B, Cornut M, et al. A genome-wide screen identifies IRF2 as a key regulator of caspase-4 in human cells. EMBO Rep. 2019;20(9):e48235.
32. Napier BA, Brubaker SW, Sweeney TE, Monette P, Rothmeier GH, Gertsvolf NA, et al. Complement pathway amplifies caspase-11-dependent cell death and endotoxin-induced sepsis severity. J Exp Med. 2016;213(11):2365-82.
33. Shi J, Zhao Y, Wang K, Shi X, Wang Y, Huang H, et al. Cleavage of GSDMD by inflammatory caspases determines pyroptotic cell death. Nature. 2015;526(7575):660-5.
34. Choe D, Lee E, Song Y, Kim SC, Jeong KJ, Palsson B, et al. CRISPRi screening reveals E. coli's anaerobic-like respiratory adaptations to gentamicin: membrane depolarization by CpxR. mSystems. 2025;10(7):e0035325.
35. To TL, Cuadros AM, Shah H, Hung WHW, Li Y, Kim SH, et al. A Compendium of Genetic Modifiers of Mitochondrial Dysfunction Reveals Intra-organelle Buffering. Cell. 2019;179(5):1222-38 e17.
36. Doench JG, Fusi N, Sullender M, Hegde M, Vaimberg EW, Donovan KF, et al. Optimized sgRNA design to maximize activity and minimize off-target effects of CRISPR-Cas9. Nature Biotechnology. 2016;34(2):184-91.
37. Xu Y, Viswanatha R, Sitsel O, Roderer D, Zhao H, Ashwood C, et al. CRISPR screens in Drosophila cells identify Vsg as a Tc toxin receptor. Nature. 2022;610(7931):349-55.
38. Li X, He L, Luo J, Zheng Y, Zhou Y, Li D, et al. Paeniclostridium sordellii hemorrhagic toxin targets TMPRSS2 to induce colonic epithelial lesions. Nat Commun. 2022;13(1):4331.
39. Lee H, Beilhartz GL, Kucharska I, Raman S, Cui H, Lam MHY, et al. Recognition of Semaphorin Proteins by P. sordellii Lethal Toxin Reveals Principles of Receptor Specificity in Clostridial Toxins. Cell. 2020;182(2):345-56 e16.
40. Tian S, Liu Y, Wu H, Liu H, Zeng J, Choi MY, et al. Genome-Wide CRISPR Screen Identifies Semaphorin 6A and 6B as Receptors for Paeniclostridium sordellii Toxin TcsL. Cell Host Microbe. 2020;27(5):782-92 e7.
41. Tzelepis K, Koike-Yusa H, De Braekeleer E, Li Y, Metzakopian E, Dovey OM, et al. A CRISPR Dropout Screen Identifies Genetic Vulnerabilities and Therapeutic Targets in Acute Myeloid Leukemia. Cell Rep. 2016;17(4):1193-205.
42. Song N, Chen L, Ren X, Waterfield NR, Yang J, Yang G. N-Glycans and sulfated glycosaminoglycans contribute to the action of diverse Tc toxins on mammalian cells. PLoS Pathog. 2021;17(2):e1009244.
43. Chang SJ, Jin SC, Jiao X, Galan JE. Unique features in the intracellular transport of typhoid toxin revealed by a genome-wide screen. PLoS Pathog. 2019;15(4):e1007704.
44. Jeon Y, Chow SH, Stuart I, Weir A, Yeung AT, Hale C, et al. FBXO11 governs macrophage cell death and inflammation in response to bacterial toxins. Life Sci Alliance. 2023;6(6).
45. Tromp AT, Van Gent M, Abrial P, Martin A, Jansen JP, De Haas CJC, et al. Human CD45 is an F-component-specific receptor for the staphylococcal toxin Panton-Valentine leukocidin. Nat Microbiol. 2018;3(6):708-17.
46. Virreira Winter S, Zychlinsky A, Bardoel BW. Genome-wide CRISPR screen reveals novel host factors required for Staphylococcus aureus alpha-hemolysin-mediated toxicity. Sci Rep. 2016;6:24242.

47. Shahi I, Llaneras CN, Perelman SS, Torres VJ, Ratner AJ. Genome-Wide CRISPR-Cas9 Screen Does Not Identify Host Factors Modulating Streptococcus agalactiae beta-Hemolysin/Cytolysin-Induced Cell Death. Microbiol Spectr. 2022;10(1):e0218621.
48. Drabavicius G, Daelemans D. Intermedilysin cytolytic activity depends on heparan sulfates and membrane composition. PLoS Genet. 2021;17(2):e1009387.
49. Sanduja P, Schmieder SS, Baddal B, Tian S, Velarde JJ, Lencer WI, et al. SLO co-opts host cell glycosphingolipids to access cholesterol-rich lipid rafts for enhanced pore formation and cytotoxicity. mBio. 2025;16(3):e0377724.
50. Quarato G, Mari L, Barrows NJ, Yang M, Ruehl S, Chen MJ, et al. Mitophagy restricts BAX/BAK-independent, Parkin-mediated apoptosis. Sci Adv. 2023;9(21):eadg8156.
51. Jami S, Deuis JR, Klasfauseweh T, Cheng X, Kurdyukov S, Chung F, et al. Pain-causing stinging nettle toxins target TMEM233 to modulate Na(V)1.7 function. Nat Commun. 2023;14(1):2442.
52. Tsui CK, Twells N, Durieux J, Doan E, Woo J, Khosrojerdi N, et al. CRISPR screens and lectin microarrays identify high mannose N-glycan regulators. Nat Commun. 2024;15(1):9970.
53. Gutmann F, Jann C, Pereira F, Johansson A, Steinmetz LM, Patil KR. CRISPRi screens reveal genes modulating yeast growth in lignocellulose hydrolysate. Biotechnol Biofuels. 2021;14(1):41.
54. Tsui CK, Barfield RM, Fischer CR, Morgens DW, Li A, Smith BAH, et al. CRISPR-Cas9 screens identify regulators of antibody-drug conjugate toxicity. Nat Chem Biol. 2019;15(10):949-58.
55. Zhu Y, Groth T, Kelkar A, Zhou Y, Neelamegham S. A GlycoGene CRISPR-Cas9 lentiviral library to study lectin binding and human glycan biosynthesis pathways. Glycobiology. 2021;31(3):173-80.
56. Morgens DW, Wainberg M, Boyle EA, Ursu O, Araya CL, Tsui CK, et al. Genome-scale measurement of off-target activity using Cas9 toxicity in high-throughput screens. Nat Commun. 2017;8:15178.
57. Lau MT, Ghazanfar S, Parkin A, Chou A, Rouaen JR, Littleboy JB, et al. Systematic functional identification of cancer multi-drug resistance genes. Genome Biol. 2020;21(1):27.
58. Rovsing AB, Thomsen EA, Nielsen I, Skov TW, Luo Y, Dybkaer K, et al. Resistance to vincristine in DLBCL by disruption of p53-induced cell cycle arrest and apoptosis mediated by KIF18B and USP28. Br J Haematol. 2023;202(4):825-39.
59. Zhong C, Jiang WJ, Yao Y, Li Z, Li Y, Wang S, et al. CRISPR screens reveal convergent targeting strategies against evolutionarily distinct chemoresistance in cancer. Nat Commun. 2024;15(1):5502.
60. Ramaker RC, Hardigan AA, Gordon ER, Wright CA, Myers RM, Cooper SJ. Pooled CRISPR screening in pancreatic cancer cells implicates co-repressor complexes as a cause of multiple drug resistance via regulation of epithelial-to-mesenchymal transition. BMC Cancer. 2021;21(1):632.
61. Su D, Feng X, Colic M, Wang Y, Zhang C, Wang C, et al. CRISPR/CAS9-based DNA damage response screens reveal gene-drug interactions. DNA Repair. 2020;87:102803.

62. Wang B, Wan AH, Xu Y, Zhang RX, Zhao BC, Zhao XY, et al. Identification of indocyanine green as a STT3B inhibitor against mushroom alpha-amanitin cytotoxicity. Nat Commun. 2023;14(1):2241.
63. Zhu Q, Ma Y, Liang J, Wei Z, Li M, Zhang Y, et al. AHR mediates the aflatoxin B1 toxicity associated with hepatocellular carcinoma. Signal Transduct Target Ther. 2021;6(1):299.
64. Zhang J, Hu S, Zhao C, Zhou Y, Zhang L, Liu H, et al. Genome-Scale CRISPR Knockout Screening Identifies BACH1 as a Key Regulator of Aflatoxin B(1)-Induced Oxidative Damage. Antioxidants (Basel). 2022;11(9).
65. Yang H, Ji X, Zhong H, Yang X, Hu D, Cai G, et al. CRISPR screening identifies protein methylation and ubiquitination modifications that modulate aflatoxin B(1) cytotoxicity. Sci China Life Sci. 2025;68(7):2121-36.
66. Lin J, Miao J, Schaefer KG, Russell CM, Pyron RJ, Zhang F, et al. Sulfated glycosaminoglycans are host epithelial cell targets of the Candida albicans toxin candidalysin. Nat Microbiol. 2024;9(10):2553-69.
67. Shi WT, Yao CP, Liu WH, Cao WY, Shao W, Liao SQ, et al. An fusaric acid-based CRISPR library screen identifies MDH2 as a broad-spectrum regulator of Fusarium toxin-induced cell death. J Hazard Mater. 2024;480:135937.
68. Xu X, Wu Y, Zhao Y, Liu A, Yi C, Zhang A, et al. Inhibition of Macrophage Pyroptosis horizontal line A New Therapeutic Strategy to Alleviate T-2 Toxin-Induced Subacute Liver Injury by Directly Competing with the Key Target. J Agric Food Chem. 2024;72(33):18670-81.
69. Lau M-T, Manion J, Littleboy JB, Oyston L, Khuong TM, Wang Q-P, et al. Molecular dissection of box jellyfish venom cytotoxicity highlights an effective venom antidote. Nature Communications. 2019;10(1):1655.
70. Du TY, Hall SR, Chung F, Kurdyukov S, Crittenden E, Patel K, et al. Molecular dissection of cobra venom highlights heparinoids as an antidote for spitting cobra envenoming. Sci Transl Med. 2024;16(756):eadk4802.
71. Fernandez-Carrasco I, Moral-Sanz J, Kurdyukov S, Monne ÈO, Hartmann LM, Magalhães Novais SC, et al. The cytotoxicity of gomesin peptides is mediated by the glycosphingolipid pathway and lipid-cholesterol interactions. Cell Death Discovery. 2025;11(1):538.
